# Assessing the Metabolic Diversity of *Streptococcus* from a Protein Domain Point of View

**DOI:** 10.1371/journal.pone.0137908

**Published:** 2015-09-14

**Authors:** Edoardo Saccenti, David Nieuwenhuijse, Jasper J. Koehorst, Vitor A. P. Martins dos Santos, Peter J. Schaap

**Affiliations:** Laboratory of Systems and Synthetic Biology, Wageningen University and Research Centre, Wageningen, The Netherlands; Beijing Institute of Microbiology and Epidemiology, CHINA

## Abstract

Understanding the diversity and robustness of the metabolism of bacteria is fundamental for understanding how bacteria evolve and adapt to different environments. In this study, we characterised 121 *Streptococcus* strains and studied metabolic diversity from a protein domain perspective. Metabolic pathways were described in terms of the promiscuity of domains participating in metabolic pathways that were inferred to be functional. Promiscuity was defined by adapting existing measures based on domain abundance and versatility. The approach proved to be successful in capturing bacterial metabolic flexibility and species diversity, indicating that it can be described in terms of reuse and sharing functional domains in different proteins involved in metabolic activity. Additionally, we showed striking differences among metabolic organisation of the pathogenic serotype 2 *Streptococcus suis* and other strains.

## Introduction

Understanding the diversity and robustness of the metabolism of bacteria is a fundamental step in the study of organisms, as these may mirror the large variety of approaches and strategies that species have taken to adapt to the environment. Nonetheless, even in well-studied bacterial model organisms, our knowledge of metabolism is far from complete [1]. Several functions, such as carbon catabolism, biosynthesis of amino acids, nucleotides, vitamins, and cofactors, are common to most bacteria and define the core metabolism. Yet, mapping the metabolic diversity in bacteria requires the characterisation of metabolic processes and functionalities that are unique to a subset of organisms. Understanding how new metabolic pathways emerge is one of the key questions in evolutionary and systems biology [2–4].

The prevailing paradigm is that new metabolic pathways, and thus new metabolic functions, arise through the exchange of enzymes from other existing metabolic networks [5]. Enzyme promiscuity plays an important role as well: this refers to the fact that many enzymes have limited substrate specificity and thus can catalyse chemical reactions other than those for which they have evolved [2], leading to the formation of small amounts of alternative products. The ensemble of metabolic pathways that can be potentially formed by these enzymes is known as the underground metabolism [3]. The role of underground reactions in the adaptation of bacterial species toward novel environments or the acquisition of novel functionalities is largely unknown. Currently, the systematic detection and characterisation of underground activities by unbiased high-throughput approaches is not feasible [2] and the prediction of phenotypic evolution, starting from (a detailed) knowledge of the underground metabolism, is still out of reach [2]. However, its characterisation is essential for understanding bacterial metabolic robustness and flexibility [6].

In this study, we set out to characterise bacterial metabolic diversity—that is, the ability of gaining (or losing) new metabolic functionalities from a protein domain perspective, investigating the working hypothesis that can explain, or at least describe, the observed metabolic diversity in terms of reuse and sharing functional domains in different proteins.

Functional domains can be defined as compact modules, characterised by structural and/or sequence conservation, folding from a single polypeptide chain [7]. The aggregate of such flexible, multifunctional and structurally independent functional building blocks has become the accepted idea of what defines a protein, replacing the idea of a rigid key–lock fashion-operating machine [8]. It has been shown that a general function of a homolog of a multi-domain protein is conserved but can be altered by modification of domain specific sites and by changing its domain context by expanding, altering or exchanging a domain module [9].

The underlying premise is that proteins were able to evolve into the flexible multifunctional entities that we know today by altering a protein’s domain architecture, either via gaining, losing, duplicating and substituting domains [10]. This results in the observation that a limited number of domains results in an incredibly large and diverse array of protein functionalities [11] as a consequence of domain promiscuity. This is a key concept for defining metabolic flexibility and diversity. Metabolic diversity of bacteria and the robustness of metabolism may limit the possibilities for using new antimicrobials [12] and hamper the prediction of new drug targets that may effectively interact with the metabolism of pathogens [13].

In this light, *Streptococci* are a particularly good study target; several streptococcal species are non-pathogenic and are active members of the commensal human microbiome [14], whereas others are able to infect a wide range of human and animal tissues [15,16], causing, for instance, not only the common strep throat but also life-threatening pathologies, such as meningitis, pneumonia, endocarditis and necrotising fasciitis. For this reason, we investigated the metabolic diversity of 121 fully sequenced *Streptococcus* strains corresponding to 29 different species.

We performed a comparative analysis of all strains by investigating their metabolic pathways on the basis of their domain architecture, characterising those metabolic pathways on the basis of the promiscuity of the constituent domains as a key factor underlying underground metabolism.

The diversity of metabolic pathways in different species and strains was defined by considering the promiscuity of domains participating in metabolic pathways that were inferred to be functional by means of an in-depth bioinformatics analysis of the *de novo* re-annotated genomes of the 121 strains. Domain promiscuity was defined by adapting existing measures based on domain abundance and versatility. The similarity of metabolic pathways from a domain architecture point of view was assessed and investigated by making use of information retrieval techniques and advanced statistical methods.

We showed that this is successful not only in capturing and describing bacterial metabolic flexibility and diversity of the different *Streptococcus* strains and species, but also in highlighting striking differences among the metabolic organisation of the highly pathogenic serotype 2 *S*. *sui* and other strains.

## Materials and Methods

### Genome sequence retrieval

**Fig 1:**
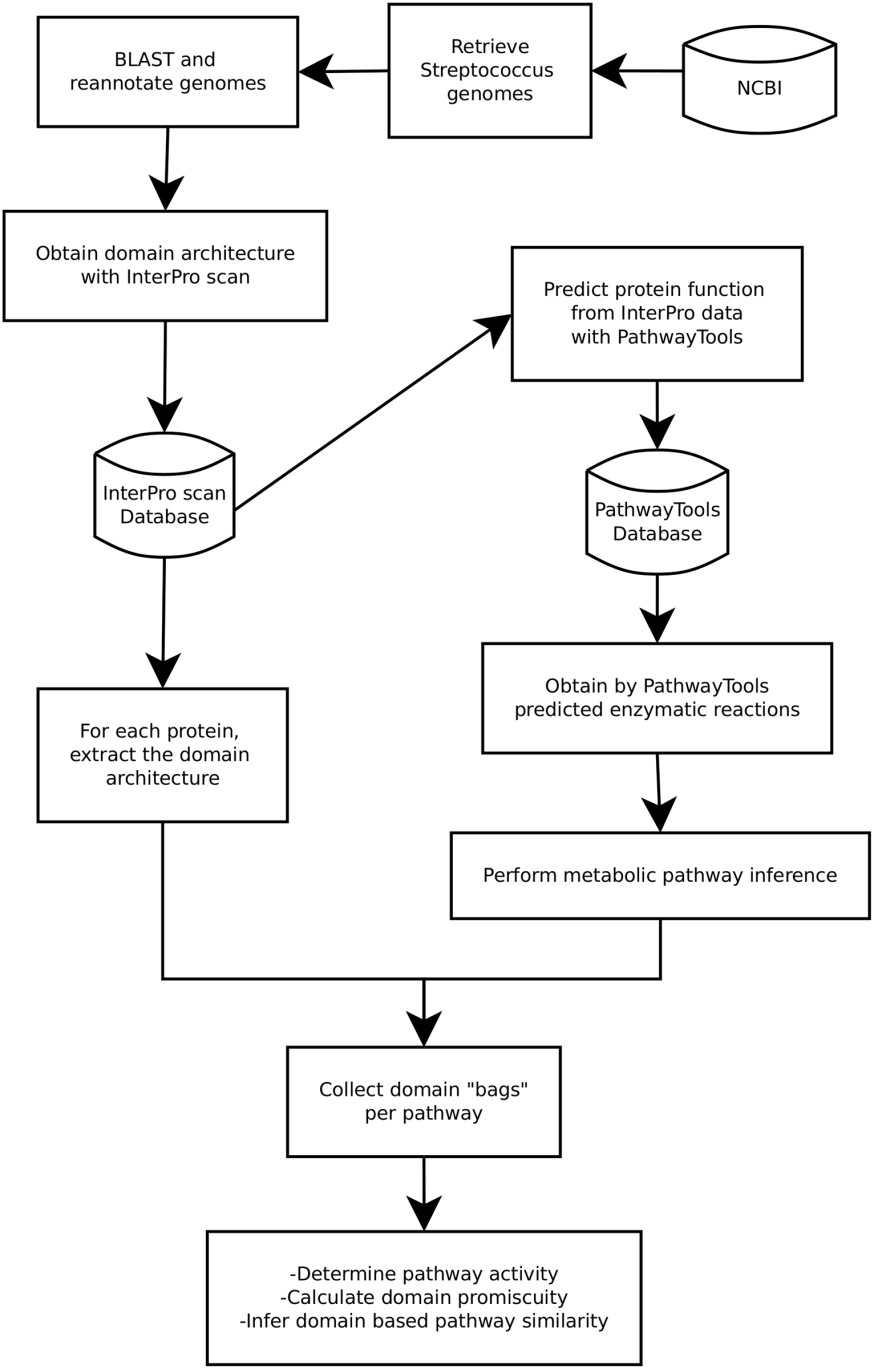
Setup of the computational pipeline for domain and pathway annotation/prediction.

Fig 1 provides an overview of the computational pipeline deployed in this study. All fully sequenced streptococcus genomes were retrieved from the NCBI FTP repository (ftp://ftp.ncbi.nlm.nih.gov/genomes/Bacteria/). A semantic data model (Resource Description Framework (RDF) [17]) was applied to convert and store Genbank files in a triplestore database using an in-house pipeline. This was done to enhance and improve the speed of performing comparative genomics. After conversion of the Genbank files into RDF, a uniform gene prediction was performed on all retrieved genomes. For gene-calling, Prodigal 2.6 was used with codon table 11 [18]. New gene predictions and the original Genbank gene predictions and annotation were stored in the semantic database.

### Domain assignment

We used InterProScan version 5 [19] to assign domain architectures to the *Streptococcus* genome sequences. InterProScan combines multiple applications [20–29], utilising a variety of algorithms to generate a consensus output [30,31]. All applications and lookup options implemented in the InterProScan tool were used, except for Panther.

A domain architecture is assigned to each open reading frame consisting of InterPro entries that are connected to the InterPro database [32]. Additionally, using InterProScan, we inferred potential Gene Ontology (GO) terms [33], EC-numbers [34], KEGG [35], MetaCyc [36] and UniPathway [37] pathway identifiers, which we used for further biological interpretation of gene function. InterProScan analysis of the 121 *Streptococci* strains was carried out using multi-threading computation with the Dutch Life Science computation grid [38].

Data obtained by InterProScan were converted to RDF using an in-house written python script implementing RDFlib (https://github.com/RDFLib/rdflib) and stored in the triplestore database. Results were retrieved using the SPARQL query language [39].

### Inference of functional metabolic pathways

Metabolic pathways available to the different *Streptococcus* strains analysed were inferred using Pathway Tools [40]. Pathways were predicted from the MetaCyc pathway database [36]. GO terms, EC number and InterPro describers were used as input to generate a Pathway/Genome Database (PGDB) containing the predicted metabolic pathways for each of the given strains.

To obtain unbiased information of the metabolic capabilities of the considered *Streptococcus* strains, we did not use the standard pathway inference implemented in Pathway Tools as it includes a gap-filling step that may bias consecutive comparative analyses. Instead, we included the Pathway Tools annotation functionality within a custom script to objectively assign MetaCyc reactions, annotated by Pathway Tools, to MetaCyc pathways. This prevents false positive pathway prediction and allows for further analysing pathway reconstruction based on domain shuffling, a functionality not present in Pathway Tools. In this way, the metabolic annotation was semi-automatically curated for considering whether or not a given metabolic pathway was available in a given strain.

### Determination of domain promiscuity

To determine domain promiscuity, we adopted and adapted the method proposed by Lee and Lee [41], which considers every metabolic pathway as an ensemble or a “bag” of domains derived from the proteins participating in a given metabolic pathway. In this approach, the promiscuity of a domain is the combined index of two quantities: the domain inverse abundance frequency *IAF* and the domain inverse versatility *IV*.

For a given domain *d*, we first considered the *IAF* defined as

$$IAF(\text{d})\;=\text{ log }\frac{p_{t}}{p_{d}}$$ (1)

Where *P*_*d*_ is the number of metabolic pathways in a given strain and *P*_*t*_ is the number of metabolic pathways that contains the domain *d*. This index accounts for how specific a domain is to certain pathways, thus it measures the importance of a domain to define a metabolic pathway.

The domain inverse versatility is defined as

$$IV(\text{d})\;=\;\frac{1}{f_{d}}$$ (2)

where *f*_*d*_ is the number of unique direct neighbours of domain *d* appearing in all metabolic pathways for a given strain. This measure is based on the rationale that domains with a high amount of different directly neighbouring domains can be found, in principle, in different architectures and should therefore be more promiscuous than would domains with a limited number of directly neighbouring domains.

The investigations by previous researchers [10] [42] showed that domain promiscuity is influenced by the abundance of a domain in the genome and thus this should be corrected for when aiming to determine the promiscuity of a domain. To do this, we introduced a corrective term, *α*(*d*), defined as

$$\alpha (d)\;=\;\frac{\eta (d)}{\eta_{\text{max}}}$$ (3)

where *η*(*d*) is the number of times that domain *d* appears considering all metabolic pathways in a strain (abundance) and *η*_*max*_ is the maximum domain abundance observed in the strain. As more abundant domains tend to be more promiscuous, *α*(*d*) eliminates the abundance effect.

Finally, the promiscuity score *π(d;s)* for domain *d* in a given strain *s* is given by the product of *IAF*, *IV* and *α*:

π(d;s) = IAF(D)  ×IV(d) × α(d) (4)

The index *π* is an inverse measure of promiscuity, thus the smaller the *π*, the larger the promiscuity of a domain.

From Eqs (1)–(5), it follows that *π(d;s)* can assume different values for the same domain when different strains are considered. This is a combined effect of the fact that the same domain can have a different number of neighbouring partners in different organisms and that the same domains can appear in a different number of pathways in different organisms. When the aim is to arrive at an absolute measure of promiscuity, a measure independent from the strain considered, we take a limiting approach, considering promiscuity as an ensemble property of a domain. For a given domain d, its absolute promiscuity π*(d)* is calculated as

$$\pi (d)\;=\;\underset{n\to \infty }{\text{lim}}\frac{1}{n}\overset{n}{\underset{i\;=\;1}{\Sigma }}\;\pi (d;n)$$ (5)

where *n* is the number of strains used for calculation of the promiscuity. The limit in Eq (5) indicates that the promiscuity of the domain *d* is calculated considering 1, 2, 3, and so on, strains until convergence. The limiting procedure was realised by resampling 100 strains for n = 1, 2, …, 121.

### Comparison of metabolic pathways

For every strain, each metabolic pathway was vectorised to a *t-*dimensional binary vector *B*_*s*_ where *t* is the total number of domains inferred by considering all 121 strains, defined as

$$B_{s}(d)\;=\;\{\begin{array}{cc} 1 & \text{if domain }d\text{ is present}\\ 0 & \text{otherwise} \end{array}$$ (6)

The 121 *B*-vectors were then collapsed into a 121 × t metabolic matrix M for further analysis.

Second, each element of *B*_*s*_ was mapped to a weighted metabolic promiscuity vector *W*_*s*_ simply defined as

$$W_{s}(d)\;=\;B_{S}(d)\;\times \;\pi (d)$$ (7)

Where *π*(*d*) and *B*_*s*_(*d*) have been defined in Eqs (5) and (6), respectively.

Given two metabolic pathways their vectorised representations *x* and *y* given by Eq (7), their (dis)similarity *D(X*,*Y)* is obtained as proposed in previous research [41]:

$$D(X,Y)=\;1\;-\frac{\sum_{d}X(d)\cdot Y(d)}{\sqrt{\sum_{d}X^{2}(d)}\cdot \sqrt{\sum_{d}Y^{2}(d)}}$$ (8)

The dissimilarity index *D*, which is based on the cosine distance between two vectors, ranges from 0 to 1; 0 indicates that the two metabolic pathways share the same domains (each with the same promiscuity) while 1 indicates that no domains are shared between the two pathways. We only consider pathways that have at least 66% of their reactions performed by a protein assigned by Pathway Tools.

### Visualisation of the (dis)similarity metabolic matrices

INDSCAL [43] was used to visualise the pathway dissimilarity matrices of the 121 *Streptococcus* strains calculated using Eq (8). The dis(similarity) matrix *S*_*k*_ for the *k*-th group can be written as

$$S_{k}\;=\;X_{k}M_{k}X_{k}^{T}$$ (9)

where *X*_*k*_ is a matrix of standardised coordinates of the objects for the *k*-th matrix and *M*_*k*_ is a diagonal matrix of weights accorded by matrix *k* to each of its dimensions. Each matrix can be seen as a set of variables (the columns of X_k_) defined on the objects. With this notation, the INDSCAL model can be written as

$$S_{k}\;=\;AW_{k}A^{T}+E_{k}$$ (10)

with *A* the *M × R* matrix of the coordinates of the objects equal for all the matrix (A is the loadings matrix) in a space with *R* dimensions and *W*_*k*_ is *R × R* diagonal matrix of the weights of matrix *k*. The elements on the diagonal of *W*_*k*_ are the coordinates for the *k*-th matrix in the *R*-dimensional space defined by INDSCAL model.

In this way, the different matrices can be then represented in an *R-*dimensional space to highlight differences and similarities. We refer the reader to previous research [43,44] for more details. To summarise, the INDSCAL model provides a methodology to represent networks in such a way as to highlight their similarities and differences, akin to principal component analysis (PCA) when applied to vectors of observations. INDSCAL model calculations have been performed using the “smacof” R-package [45]. The INDSCAL models were fitted with five components as a good compromise between fit and model complexity.

## Results and Discussion

### Re-annotation of *Streptococcus* genomes

**Fig 2:**
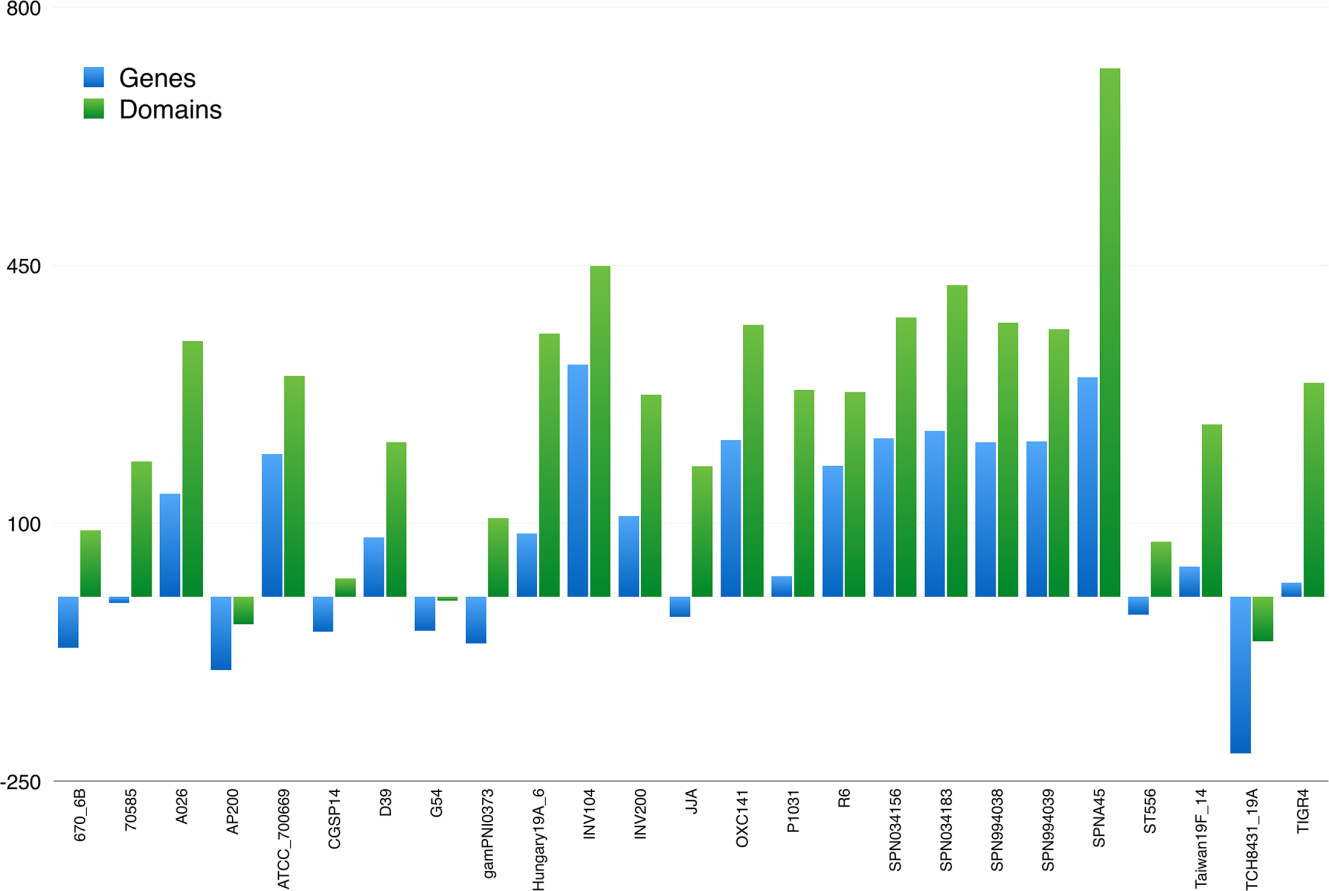
Differences in gene and domain counts between deposited and ex-novo annotation for *Streptococcus pneumoniae*. Full chart for all 121 strains is given in S1 Fig.

Public gene databases are growing exponentially and double in size approximately every 10 months [46], implying that the majority of the data is less than a year old. The genomes analysed here date back as early as 2000, and before proceeding with further analysis, we annotated *ex-novo* the 121 genomes to guarantee a uniform up-to-date annotation by applying the workflow depicted in Fig 1. As shown in Fig 2, we found that the deposited GenBank annotation files were, for the most part, outdated and were thus unsuitable for a comparative study.

### Inference of available metabolic pathways to *Streptococcus*

**Fig 3:**
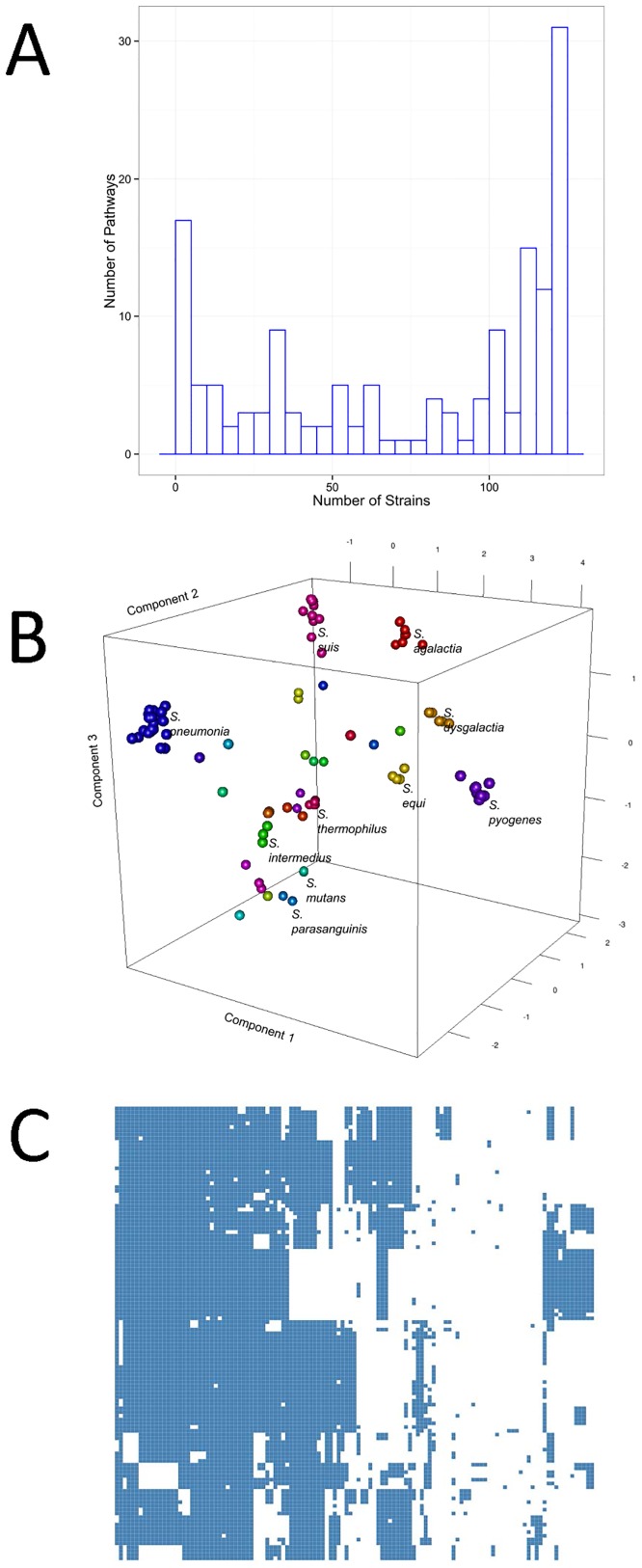
A) Distribution of functional pathways across different strains B) Score plot of principal component analysis performed on the metabolic diversity matrix. C) Hierarchical clustering performed on the metabolic diversity matrix. Strains cluster on the basis of their capability of performing certain metabolic functions.

Pathway reconstruction based on the metabolic reaction assignment by Pathway Tools resulted in 298 pathways available to *Streptococcus*. Of the inferred pathways, 27 appear in all strains. Fig 3 Panel A shows a histogram of the distribution of active pathways across the 121 *Streptococcus* strains considered. A complete list of all of the inferred pathways is given in S1 Table.

Those core pathways are involved in base cofactor production, base metabolism and cell wall/membrane metabolism: these functionalities constitute the base of bacterial metabolism and, not surprisingly, are found in all *Streptococcus* strains. Several pathways are involved in the biosynthesis of ATP, GTP, UTP, S-adenosyl-L-methionine and amino-acid conversions from one amino acid to another, which are the basic needs of any living organism. Other core pathways are involved in the metabolism of the compounds related to energy, carbon and nitrogen source utilisation, such as degradation of mannose and fermentation of pyruvate. Finally, some core pathways pertain to the production of cell wall compounds, such as fatty acids, peptidoglycans and lipopolysaccharides.

Fig 3 Panel B shows the score plot of principal component analysis performed on the metabolic diversity matrix. Strains clearly cluster according to the species, indicating that strains belonging to the same species share similar patterns of functional metabolic pathways. Fig 3 Panel C shows hierarchical clustering performed on the metabolic diversity matrix. It appears that the strains can be clustered on the basis of their metabolic (dis)similarity, which is on the basis of their capability of performing certain metabolic functions. Moreover, blocks of metabolic pathways (and thus metabolic functions) appear to be lacking in certain strains.

A striking example of missing metabolic activity is found in the species S. *pyogenes*: none of the 19 strains have the metabolic capacity to perform Glutamate degradation I, Glutamate degradation X, Glutamate biosynthesis II and Glutamate biosynthesis III, in comparison to other *Streptococcus* species where these pathways are always present. This finding is consistent with results from metabolic modelling [47] and with fermentation experiments that reported the inability of *S*. *pyogenes* to ferment glutamate [48]: this indicates that other (unknown) variants of this pathway also may not be functional. This is an interesting observation since nitrogen metabolism via glutamine and glutamate is related to pathogenicity in other *Streptoccus* species [49], and it is remarkable that S. *pyogenes* does not ferment glutamate [48] despite its severe pathogenicity. On the other hand, it can be observed that, with small exceptions, the S. *pneumonia* strains are the only *Streptococci* equipped with homospermidine biosynthesis and pyruvate oxidation pathways, which have been found to be involved in pathogenicity mechanisms [50] and in the counteraction of oxidative stress [51].

### Domain analysis

Functional domains were inferred using the domains of the gene products active in the metabolic reactions, as inferred in Pathway Tools. Considering the 121 *Streptococcus* strains and the 298 active metabolic pathways, we obtained a collection of 881 functional domains. A complete list of the domains with the associated InterPro identifier is given in S2 Table.

### Domain promiscuity analysis

Several indices have been proposed to describe promiscuity [41,52–55], taking into account different domain characteristics. However, there is general agreement about the fact that a fundamental determinant of promiscuity is the tendency of a domain to co-occur with other domains. Co-occurrence is somehow a loose concept: co-occurring domains have been defined as domains occurring in the same protein [56], domains occurring in a microenvironment of three domains [53] or domains that directly neighbour the domain in the protein sequence [41,54,55] [5]. We chose the latter interpretation of co-occurrence, taking the number of unique directly-neighbouring domains as the co-occurrence factor, a choice also supported by the observation that there is a higher tendency for domains to be gained or lost at the *C*- and *N*-terminal positions in the protein [57].

The promiscuity *π* of a domain was defined as the product of the inverse versatility *IV* and the inverse abundance frequency *IAF*, as detailed in the Material and Methods section, Eqs (1) to (5). The *IV* describes the propensity of a domain to co-occur with other domains within a protein (and thus with other domains in a certain metabolic pathway). Table 1 gives a list of domains ranked according to the number of observed neighbours. For the P-loop containing nucleoside triphosphate hydrolase (NTPase) domain IPR027417, we observed more than 140 different neighbouring domains. This domain is the most prevalent domain of the several distinct nucleotide-binding protein folds; the most common reaction catalysed by enzymes of the P-loop NTPase fold is the hydrolysis of the beta-gamma phosphate bond of a bound nucleoside triphosphate (NTP). Another highly promiscuous domain is the ABC transporter, belonging to the ATP-Binding Cassette (ABC) superfamily, which uses the hydrolysis of ATP to energise diverse biological systems. Its major function is to provide essential nutrients to bacteria and it is found only in prokaryotes. On the contrary, Table 2 shows domains with the least number of neighbours across strains. For instance, IPR001754 describes an Orotidine 5'-phosphate decarboxylase domain and IPR001342 represents the homoserine dehydrogenase catalytic domain. These lowly promiscuous domains mostly exhibit specialized functionality and are not encountered in many enzymes.

**Table 1:** Top 10 more promiscuous domains with respect to the number of different neighbouring domains.

InterPro Identifier	Average domain count	InterPro description
IPR027417	141	P-loop containing nucleoside triphosphate hydrolase
IPR003439	92	ABC transporter-like
IPR011527	32	ABC transporter type 1, transmembrane domain
IPR001564	15	Nucleoside diphosphate kinase
IPR008334	12	5'-Nucleotidase, C-terminal
IPR029052	12	Metallo-dependent phosphatase-like
IPR026459	11	Ribonucleotide reductase, class 1b, subunit NrdE
IPR009078	8	Ferritin-like superfamily
IPR026023	8	Ribonucleotide reductase small subunit, prokaryotic
IPR001645	7	Folylpolyglutamate synthetase

**Table 2:** Top 10 least promiscuous domains with respect to the number of different neighbouring domains.

InterPro Identifier	Average domain count	InterPro description
IPR000120	< 1	Amidase
IPR000191	< 1	DNA glycosylase/AP lyase
IPR000192	< 1	Aminotransferase class V domain
IPR001093	< 1	IMP dehydrogenase/GMP reductase
IPR001342	< 1	Homoserine dehydrogenase, catalytic
IPR001518	< 1	Argininosuccinate synthase
IPR001754	< 1	Orotidine 5'-phosphate decarboxylase domain
IPR001986	< 1	Enolpyruvate transferase domain
IPR003733	< 1	Thiamine phosphate synthase
IPR004188	< 1	Phenylalanine-tRNA ligase, class II, N-terminal

**Fig 4:**
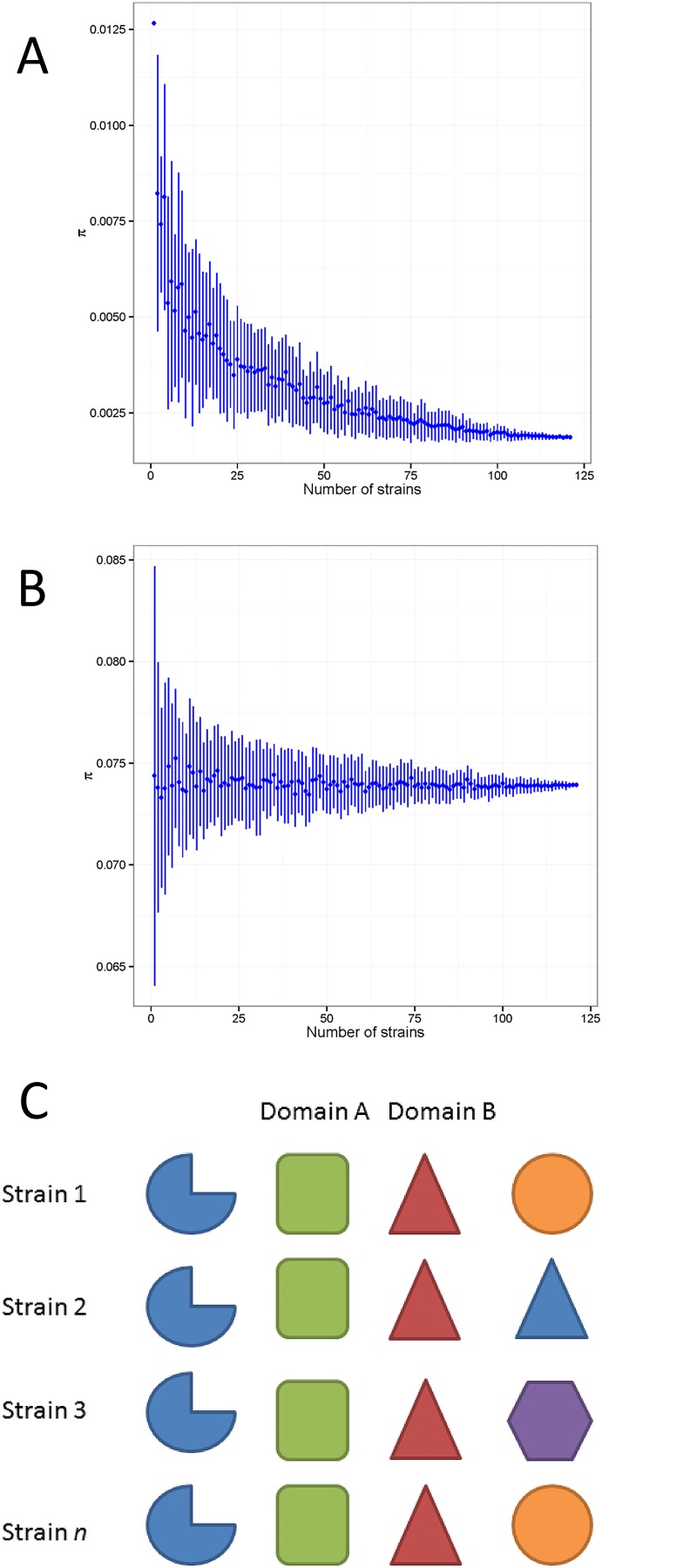
Two domains IPR000421 and IPR000115 (Panels A and B, respectively) exemplify the effect of sample size on the domain promiscuity definition. Domain A shows a decrease in promiscuity depending on the number of strains used for calculation. Domain B has a stable promiscuity, independent of the number of strains used for calculation. Panel C shows a graphical illustration of the concept of absolute promiscuity. In this cartoon, domain A is absolutely promiscuous as it has always the same neighbouring partners, which is not the case of domain B.

The *IAF* accounts for how a given domain is specific to a given metabolic pathway in a certain strain. It is clear by definition that given a domain, both *IV* and *IAF*, and in consequence *π*, are strain-specific measures of promiscuity as *IV* and *IAF* depend on the number of proteins and pathways in a given strain, respectively. Surprisingly, for most of the domains, promiscuity is strongly strain-specific and, as shown in Fig 4 Panel A, it depends on the number neighbouring domains, which is different for different strains. Table 3 shows a list of domains ranked according to the number of metabolic pathways where they occur. It can be noted that this seems to not be correlated with the number of neighbours possessed by a domain; this justifies the definition of domain promiscuity as the product of *IV* and *IAF*.

**Table 3:** Top 10 more promiscuous domains with respect to the number of different metabolic pathways they appear in.

InterPro Identifier	Number of pathways domain occurs in	InterPro description
IPR001564	10	Nucleoside diphosphate kinase
IPR027417	9	P-loop containing nucleoside triphosphate hydrolase
IPR026456	5	Glycosyltransferase AglJ
IPR013785	4	Aldolase-type TIM barrel
IPR012162	4	Polyribonucleotide nucleotidyltransferase
IPR011268	4	Purine nucleoside phosphorylase
IPR004468	4	CTP synthase
IPR009078	4	Ferritin-like superfamily
IPR026023	4	Ribonucleotide reductase small subunit, prokaryotic
IPR004402	4	Purine nucleoside phosphorylase DeoD-type

To arrive at a measure of promiscuity that is strain-independent, we take a limiting approach, considering promiscuity as an ensemble property of a domain. We observed that for a large number of domains, the variation of *π* rapidly decreases and converges towards smaller values (and thus higher promiscuity) when a sufficiently large number of organisms are considered, as can expected.

Although domains may have low specificity, the number of possible biological functions that can be performed is limited as the number of possible partners is also limited. Decreasing in promiscuity, as a function of the number of strains, is a consequence of the fact that more domain configurations and functions are taken into account when more strains are added. The plateau reached by promiscuity curves indicates that no more information is gained by adding more strains and that possibly all biologically active domain configurations in streptococcus have been considered.

Fig 4 Panel A shows the case of domain IPR000421. Interestingly enough, this is a Coagulation factor 5/8 C-terminal type domain, appearing in more than 5000 bacteria. It is a domain-like galactose binding with specific binding activity that may serve for cell adhesion in bacteria. More interesting are those domains for which *π* remains stable regardless of the number of organisms considered. Fig 4 Panel B shows the case of domain IPR000115, Phosphoribosylglycinamide synthetase, which catalyses the second step in the *de novo* biosynthesis of purine. We call these domains “absolutely promiscuous domains”; by definition of Eqs (1) to (4), it is assumed that an absolutely promiscuous domain has (on average) the same number of neighbouring partners and appears in the same number of metabolic pathways irrespective of which strain is considered. This is consistent with the observation that IPR000115 belongs to an operon and thus has a fixed number of partners. The concept of absolute promiscuity is exemplified graphically in Fig 4 Panel C. (see caption for more details).

The shape of the *π* curves determines whether a domain is promiscuous or absolutely promiscuous. We explored behaviour of the promiscuity by fitting a straight line to the logarithm of *π* curves. The value of π when all species are considered is taken as the actual measure of the promiscuity of a given domain: the larger the π, the smaller the domain’s promiscuity.

**Fig 5:**
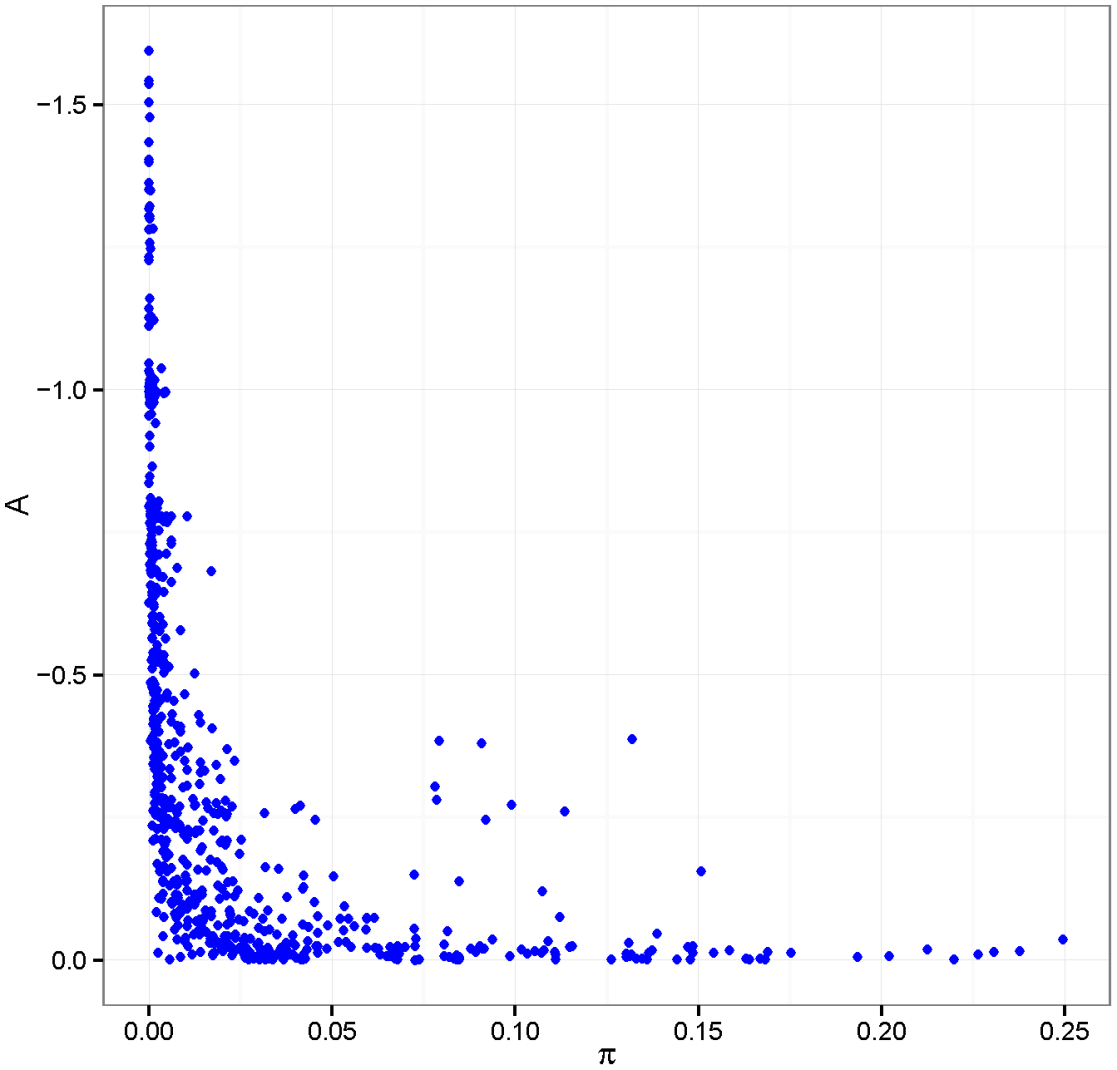
Scatterplot of A = 1/a versus the promiscuity π for every domain. Domains with smaller slope *a*, and thus larger *A*, tend to be absolutely promiscuous. The intercept gives a measure of the degree of promiscuity: the larger the *p*, the smaller the promiscuity.

The inverse *A = 1/a* of the slope *a* of the fitted line provides a measure of the absoluteness of the promiscuity of a given domain. Domains with smaller slope *a*, and thus larger *A*, tend to be absolutely promiscuous. To explore if a relationship exists between absolute promiscuity and promiscuity value, we plotted *A* versus *π* in the scatter plot given in Fig 5. Most of the domains show high promiscuity (low *π)* and low absoluteness, indicating strain-specific promiscuity. For several domains, we observe low promiscuity (high *π*) and high absoluteness. For the remaining domains, we observe a large dynamic range of the index *π*, but small absoluteness.

This observation seems to contrast with the intuition that promiscuity should be a global property of a domain rather than a local one. The combination of domains with different structures and functions within the multi-domain proteins is a major mode of creation and modulation of molecular functionality [42,55]; the observed strain-specific promiscuity points to the fact that evolution and the re-arrangement of domain architectures follow strains-specific patterns in *Streptococcus* In the eukaryotes domain, promiscuity has been found to be a volatile, relatively fast-changing feature of eukaryotic proteins, with few domains remaining promiscuous throughout the evolution of eukaryotes [55]. We observed a similar behaviour in *Streptococcus* where a smaller fraction of domains remain promiscuous in all strains; this could be linked to the great extent of bacterial flexibility and to their capacity for rapidly adapting to changing environmental conditions by re-modulation of underground metabolism.

### Metabolic diversity within the genus

Comparing the metabolism of different species based only on the presence and absence of pathways provides only a limited view of their metabolic flexibility. Looking to metabolic pathways from a domain point of view, taking into account the promiscuity of the constituent domains, can provide insights on how and to what extent domains are reused in different metabolic pathways, thus providing an indication of metabolic flexibility and adaptability across different strains.

To analyse the domain architecture of a metabolic pathway, we considered a metabolic pathway as an ensemble of domains obtained from collecting the entire domain architectures of the proteins involved in the metabolic reactions in the pathway. A given metabolic pathway is then described in terms of the promiscuity of the protein domains involved, as described by Eqs (6)) and (8).

Because domain promiscuity is strain-dependent and domains show high variability between different strains, as previously discussed (see Eqs (1) to (4)), we therefore used absolute promiscuity, as defined by Eq (5), to perform an unbiased comparison of (dis)similarity between different metabolic pathways in terms of domain promiscuity. The (dis)similarity between different pathways *X* and *Y* was calculated using the distance measure given in Eq (8).

**Fig 6:**
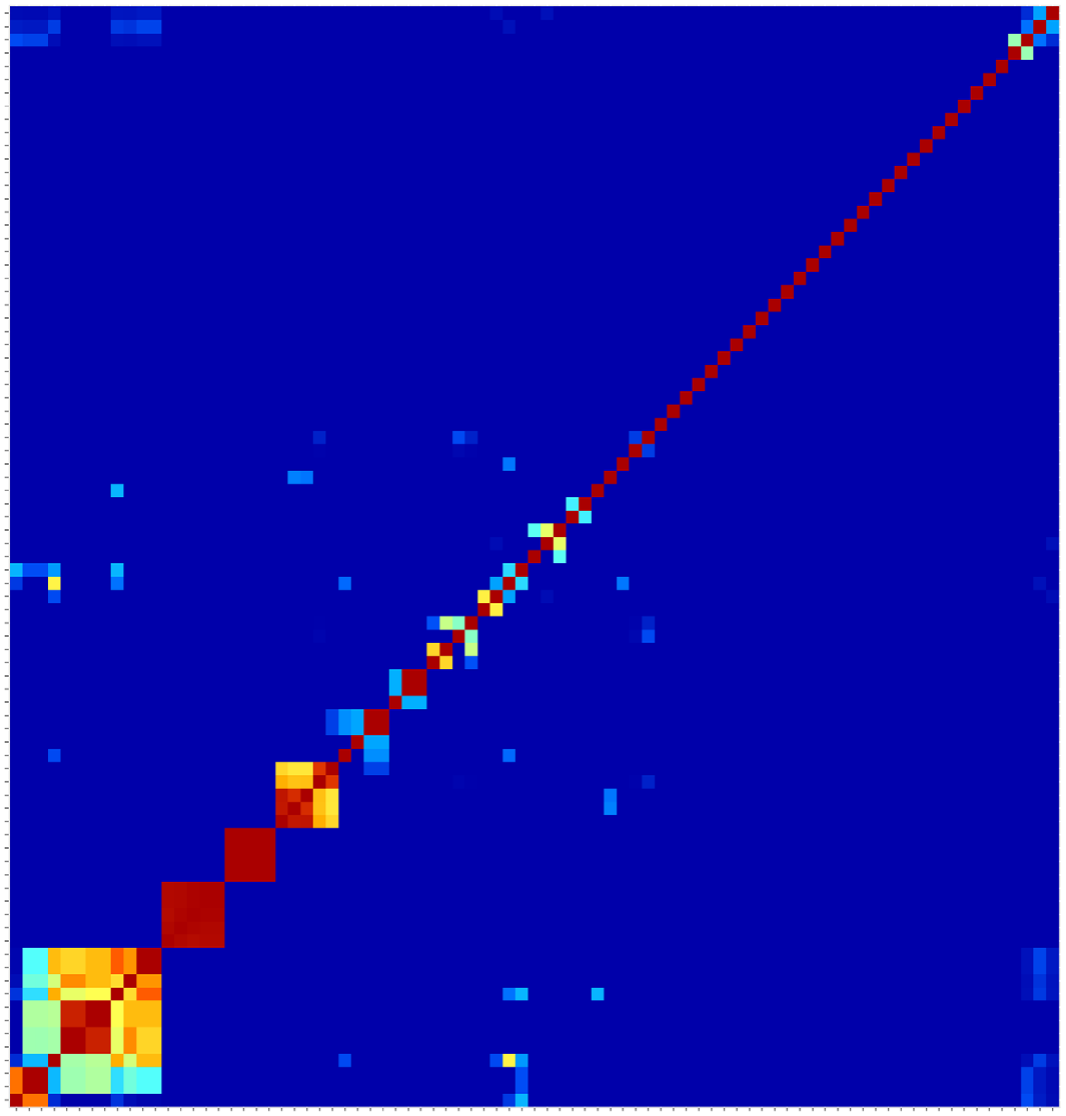
Heat map of the domain-based comparison of pathways for a single strain of *Streptococcus suis*.

For every *Streptococcus* strain, we calculated *D(X*,*Y)* for each possible pair of functional metabolic pathways obtained in a squared domain-based (dis)similarity matrix. Fig 6 shows a heat map representation of the (dis)similarity for *S*. *pneumonie*. There are blocks of pathways with higher domain-based similarity, indicating distinct biological pathways that possess similar domain architecture. For instance, we observed that deoxyribonucleotide synthesis pathways and the glutamate metabolism pathways cluster together as they are functionally related. However, pathways that are functionally more distant also show high domain-based similarity, for instance the pathways for glycerol and ethanol degradation.

Blocks of similar pathways may suggest the existence of sets of domain cores that act as building blocks for multiple metabolic pathways that are formed by incorporation and/or loss of domains, conferring to each species its metabolic individuality and flexibility.

We observed that pathways with new functionality could be generated from existing pathways by the addition of one or more complete enzymes. However, the acquisition of an entirely new enzyme is a large evolutionary step for an organism. The question arises whether the remodulation of existing domain architectures within an organism would be sufficient for generating novel or alternative metabolic capabilities without the introduction of new enzymes. This would be consistent with the observation that functions of the individual domains in the multidomain proteins combine to produce their overall functions [7–9]. Preliminary results show that dozens of reactions could be performed by altering fewer than 25% of the protein domains. Unfortunately, limitations in the domain annotation obtained by InterProScan hamper the reconstruction of alternative metabolic pathways for such reactions and further work would be required for this task.

### Metabolic diversity across different species

We conclude by comparing the (dis)similarity of metabolic pathways within different *Streptococcus* species, through the calculations of *D(X*,*Y)* for each possible pair of the 27 metabolic pathways that were found active in all of the 121 *Streptococcus* strains. Each strain results in a 27 × 27 symmetric matrix bearing the (dis)similarities between the 27 metabolic pathways described in terms of their domain promiscuity composition; these matrices can be visualised as the one given in Fig 6. It is evident that some pathways are more similar in terms of domain composition/promiscuity than are others.

What is of interest here is to investigate whether these dissimilarities among pathways are the same for all of the strains or are different between species. The INDSCAL modelling allows for a synthetic representation of the (dis)similarities among the core pathways as each matrix becomes a point in the INDSCAL space; the closer the point in space, the more similar the matrices are and thus, the more similar the domain architecture of the core domains.

**Fig 7:**
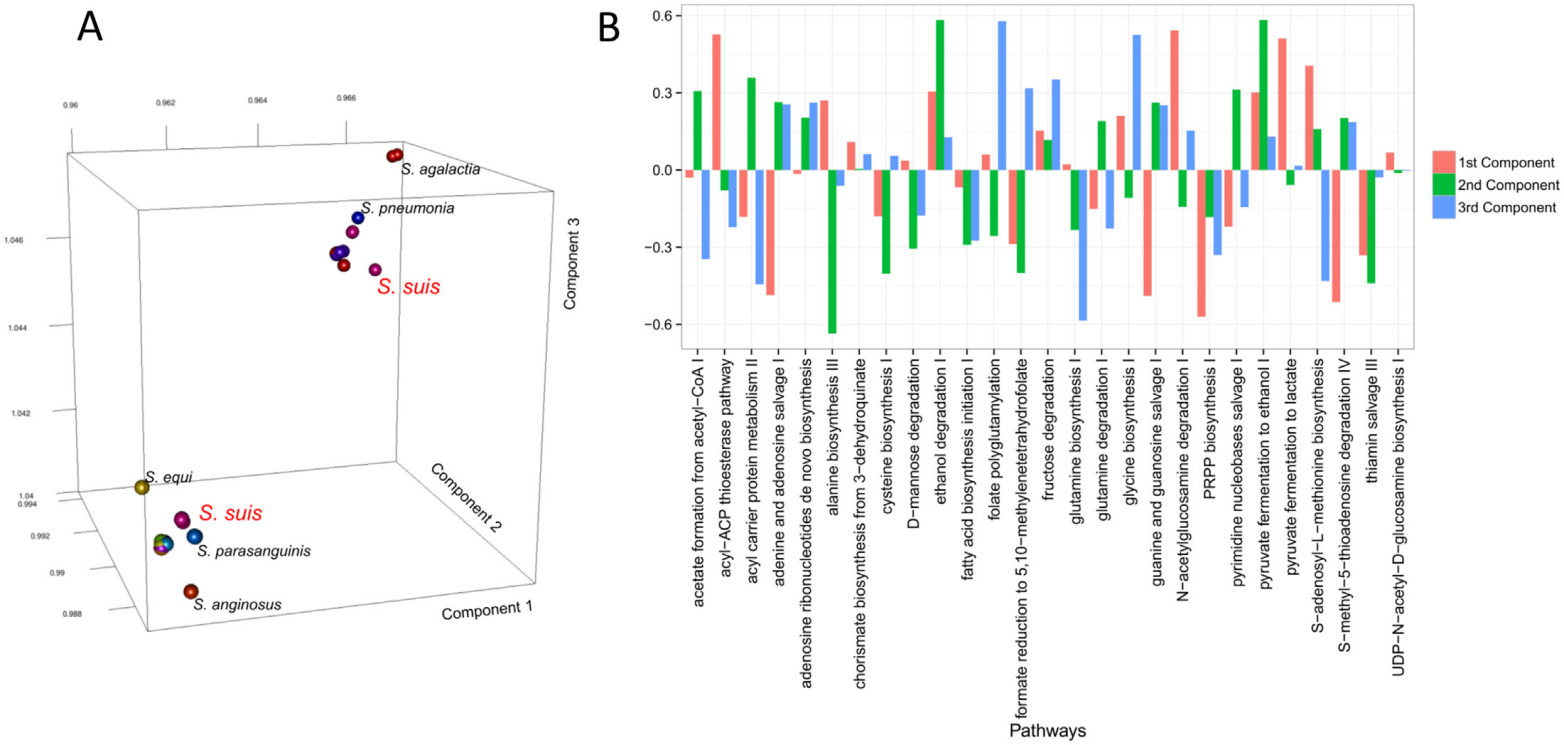
A) 3D score plot for the INDSCAL model on the (dis)similarity matrices of the 27 core metabolic pathways of the 121 *Streptococcus* strains. B) Loadings of the INDSCAL model.

When plotted on a three-dimensional space, we observe that the 121 species naturally cluster in two groups (see Fig 7 Panel A). One is formed by *S*. *agalactiae* (nine strains), *S*. *pyogenes* (19 strains) and *S*. *suis* (11 strains), and the other contains *S*. *pneumoniae* (26 strains), *S*. *suis* (six strains), *S*. *thermophilus* (six strains) and other minor strains. This clustering clearly indicates that the way the core metabolic pathways differ among each other is different, for instance, in *S*. *pyogenes* and in *S*. *peneumoniae*. In other words, it seems that for the strains analysed, the 27 core pathways appear in two major variants (corresponding to the two larger clusters) with smaller variants among the member of the same cluster. It should be remembered that in our approach, a vector whose elements are the absolute domain promiscuity represents each metabolic pathway. As domain promiscuity filters out strain-specificity, as previously discussed, this means that the core domains differ not only because of promiscuity, but also because of domain arrangements.

The loading of the INDSCAL model can be inspected to highlight which metabolic pathways are more important for explaining the observed clustering, thus suggesting which metabolic pathways differentiate between the different strains. As shown in Fig 7 Panel B, the first component shows high loadings for pathways pointing to nucleotides synthesis/degradation (PRPP biosynthesis, guanine/guanosine salvage I, adenine/adenosine salvage I) and fatty acids biosynthesis (acyl-ACP thioesterase and N-acetyl-glucosamine degradation I). The second component has high loadings on pyruvate to ethanol fermentation I, ethanol degradation and alanine biosynthesis III. The third INDSCAL component is characterised by high loadings associated with glycine and glutamine biosynthesis, 5-adenosyl L-methionine biosynthesis and folate polyglutamylation, thus describing amino acids biosynthesis.

The higher loadings associated with these pathways indicate that the considered strains mostly differ in the relationships between these pathways and it is the differences between these pathways that cause the observed clustering between strains and species.

### Metabolic diversity of the *S*. *suis* strains

It is interesting to note that the *S*. *suis* species appears in both clusters, suggesting the existence of two different subspecies. On the basis of this, we performed a more in-depth analysis of the core metabolism of *S*. *suis* strains, which account for 40 functional pathways. When the same analysis is performed only on the *S*. *suis* strains, we observed that the serotype 2 strains cluster separately from strain with serotypes ½, 1, 3, 7, 9 and 14. It has been observed that serotype 2 *S*. *suis* strains are phylogenetically distinct from other serotypes [58], although we observed a sharper separation with respect to serotype.

Serotipisation is based on the presence of specific antigenic polysaccharides and the biosynthesis of capsular polysaccharides (cprs) requires an extremely complex pathway [59,60]. By comparing the metabolic pathways across the *S*. *suis* strains, we observed that several pathways are functional in *S*. *suis 2*, but not in the other serotypes and vice versa; in particular, we found that the UDP-α-D-glucuronate biosynthesis pathway (a precursor to capsular polysaccharides biosynthesis) is not present in *S*. *suis 2* where an alternative UDP-N-acetylmuramoyl-pentapeptide biosynthesis I is found. The presence/absence of these two pathways discriminates between serotype 1 and other serotypes; nonetheless, we obtained separation of the serotype on the basis of the shared common pathways in which these pathways are not present. This indicates that different biological functions other than biosynthesis of capsular polysaccharides may be mirrored by serotype and that a comparison of different strains based on domain architecture may be more sensitive in highlighting biological and functional differences.

*S*. *suis* 2 is the most virulent form of the bacteria and is most often isolated in infected animals [61]; it is also an emergent zoonotic agent able to infect mammals and humans through skin wounds during contact with pigs or pig products [61]. Our analysis shows that the domain architecture of the metabolic pathways of *S*. *suis 2* is markedly different from that of other serotypes. Exploring differences among the core metabolic pathways may provide hints for understanding the mechanism underlying the pathogenicity of *S*. *suis 2*, providing novel tools for diagnostic purposes and for epidemiological and transmission studies.

## Conclusions

Bacterial species are able to adapt to different environments and conditions as a result of their metabolic flexibility. In this paper, we characterised proteins domains in terms of promiscuity, defined by adapting existing measures based on domain abundance and versatility. Subsequently, metabolic pathways that were inferred to be active in 121 *Streptococcus* strains were represented in terms of the domains participating in the metabolic reactions. This resulted in a numerical matrix representation of the metabolic capabilities of the considered strains, which allowed for in-depth statistical analysis.

Through this approach, we could capture not only bacterial metabolic flexibility but also species diversity, indicating that it can be described in terms of reuse and sharing functional domains present in different proteins. We highlighted metabolic differences between species and even between strains within the same species, as is the case with the metabolic organisation of the pathogenic serotype 2 *Streptococcus suis* and other strains. Our study indicates that comparative analysis of different organisms on the basis of their domain-based architecture can prove to be a complementary tool for comparative genomics.

## Supporting Information

S1 Fig Gene counts for the 121 bacterial strains. (PDF)

S1 Table A complete list of all of the inferred pathways. (XLSX)

S2 Table A complete list of the domains with the associated InterPro identifier. (XLSX)
