# Supplementary material for: Assessing the Metabolic Diversity of Streptococcus from a Protein Domain Point of View
Source: PLoS One. 2015 Sep 14;10(9):e0137908. doi: 10.1371/journal.pone.0137908 (PMC4569324; doi:10.1371/journal.pone.0137908)
Supplement: S1 Fig — (PDF) [file pone.0137908.s001.pdf]

Table 1

Genomes		Genes	Domains
Streptococcus_agalactiae_09mas018883_uid208674	1	-32	12
Streptococcus_agalactiae_2_22_uid202215	2	309	940
Streptococcus_agalactiae_2603V_R_uid57943	3	-21	135
Streptococcus_agalactiae_A909_uid57935	4	74	174
Streptococcus_agalactiae_GD201008_001_uid175780	5	19	152
Streptococcus_agalactiae_ILRI005_uid208676	6	-50	-2
Streptococcus_agalactiae_ILRI112_uid208675	7	-47	20
Streptococcus_agalactiae_NEM316_uid61585	8	46	56
Streptococcus_agalactiae_SA20_06_uid178722	9	96	368
Streptococcus_anginosus_C1051_uid218003	10	120	216
Streptococcus_anginosus_C238_uid218004	11	220	421
Streptococcus_constellatus_pharyngis_C1050_uid218002	12	185	314
Streptococcus_constellatus_pharyngis_C232_uid217998	13	177	299
Streptococcus_constellatus_pharyngis_C818_uid218001	14	178	312
Streptococcus_dysgalactiae_equisimilis_167_uid222822	15	3	16
Streptococcus_dysgalactiae_equisimilis_AC_2713_uid178644	16	-88	-24
Streptococcus_dysgalactiae_equisimilis_ATCC_12394_uid161979	17	101	420
Streptococcus_dysgalactiae_equisimilis_GGS_124_uid59103	18	8	64
Streptococcus_dysgalactiae_equisimilis_RE378_uid176684	19	222	438
Streptococcus_equi_4047_uid59259	20	171	418
Streptococcus_equi_zooepidemicus_ATCC_35246_uid162155	21	-84	186
Streptococcus_equi_zooepidemicus_MGCS10565_uid59263	22	-31	215
Streptococcus_equi_zooepidemicus_uid59261	23	89	125
Streptococcus_gallolyticus_ATCC_43143_uid162103	24	36	122
Streptococcus_gallolyticus_ATCC_BAA_2069_uid63617	25	-31	15
Streptococcus_gallolyticus_UCN34_uid46061	26	32	41
Streptococcus_gordonii_Challis_substr__CH1_uid57667	27	4	106
Streptococcus_I_G2_uid224251	28	28	169
Streptococcus_I_P16_uid224252	29	-21	28
Streptococcus_infantarius_CJ18_uid87033	30	186	447
Streptococcus_iniae_SF1_uid206041	31	-19	72
Streptococcus_intermedius_B196_uid218000	32	71	101
Streptococcus_intermedius_C270_uid217999	33	65	87
Streptococcus_intermedius_JTH08_uid168614	34	139	238
Streptococcus_lutetiensis_033_uid213397	35	103	391
Streptococcus_macedonicus_ACA_DC_198_uid81631	36	155	447
Streptococcus_mitis_B6_uid46097	37	47	50
Streptococcus_mutans_GS_5_uid169223	38	16	111
Streptococcus_mutans_LJ23_uid162197	39	-51	-10
Streptococcus_mutans_NN2025_uid46353	40	-23	1089
Streptococcus_mutans_UA159_uid57947	41	-63	23
Streptococcus_oligofermentans_AS_1_3089_uid201429	42	42	89
Streptococcus_oralis_Uo5_uid65449	43	-40	-24
Streptococcus_parasanguinis_ATCC_15912_uid49313	44	-40	-8
Streptococcus_parasanguinis_FW213_uid163997	45	-10	24
Streptococcus_parauberis_KCTC_11537_uid67355	46	485	721
Streptococcus_pasteurianus_ATCC_43144_uid68019	47	142	308
Streptococcus_pneumoniae_670_6B_uid52533	48	-69	90
Streptococcus_pneumoniae_70585_uid59125	49	-8	184
Streptococcus_pneumoniae_A026_uid226114	50	140	347
Streptococcus_pneumoniae_AP200_uid52453	51	-99	-37
Streptococcus_pneumoniae_ATCC_700669_uid59287	52	194	300
Streptococcus_pneumoniae_CGSP14_uid59181	53	-47	25
Streptococcus_pneumoniae_D39_uid58581	54	81	210
Streptococcus_pneumoniae_G54_uid59167	55	-46	-5
Streptococcus_pneumoniae_gamPNI0373_uid175861	56	-63	107
Streptococcus_pneumoniae_Hungary19A_6_uid59117	57	86	357
Streptococcus_pneumoniae_INV104_uid162039	58	315	449
Streptococcus_pneumoniae_INV200_uid162035	59	110	274
Streptococcus_pneumoniae_JJA_uid59121	60	-27	177
Streptococcus_pneumoniae_OXC141_uid162037	61	213	369
Streptococcus_pneumoniae_P1031_uid59123	62	28	281
Streptococcus_pneumoniae_R6_uid57859	63	178	278
Streptococcus_pneumoniae_SPN034156_uid197185	64	215	379
Streptococcus_pneumoniae_SPN034183_uid197186	65	225	423
Streptococcus_pneumoniae_SPN994038_uid197187	66	210	372
Streptococcus_pneumoniae_SPN994039_uid197188	67	211	363
Streptococcus_pneumoniae_SPNA45_uid174986	68	298	717
Streptococcus_pneumoniae_ST556_uid162191	69	-24	75
Streptococcus_pneumoniae_Taiwan19F_14_uid59119	70	41	234
Streptococcus_pneumoniae_TCH8431_19A_uid49735	71	-212	-60
Streptococcus_pneumoniae_TIGR4_uid57857	72	19	290
Streptococcus_pseudopneumoniae_IS7493_uid71153	73	-67	84
Streptococcus_pyogenes_A20_uid178106	74	-60	-1
Streptococcus_pyogenes_Alab49_uid162171	75	-12	56
Streptococcus_pyogenes_HSC5_uid212978	76	9	96
Streptococcus_pyogenes_M1_476_uid193766	77	195	413
Streptococcus_pyogenes_M1_GAS_uid57845	78	84	190
Streptococcus_pyogenes_Manfredo_uid57847	79	66	208
Streptococcus_pyogenes_MGAS10270_uid58571	80	-97	-21
Streptococcus_pyogenes_MGAS10394_uid58105	81	-47	-35
Streptococcus_pyogenes_MGAS10750_uid58575	82	-85	-36
Streptococcus_pyogenes_MGAS15252_uid158037	83	-32	45
Streptococcus_pyogenes_MGAS1882_uid158061	84	-16	52
Streptococcus_pyogenes_MGAS2096_uid58573	85	-83	-18
Streptococcus_pyogenes_MGAS315_uid57911	86	21	31
Streptococcus_pyogenes_MGAS5005_uid58337	87	-81	-29
Streptococcus_pyogenes_MGAS6180_uid58335	88	-70	-27
Streptococcus_pyogenes_MGAS8232_uid57871	89	54	100
Streptococcus_pyogenes_MGAS9429_uid58569	90	-121	-39
Streptococcus_pyogenes_NZ131_uid59035	91	34	558
Streptococcus_pyogenes_SSI_1_uid57895	92	26	134
Streptococcus_salivarius_57_I_uid162151	93	8	134
Streptococcus_salivarius_CCHSS3_uid70481	94	-23	-22
Streptococcus_salivarius_JIM8777_uid162145	95	-8	-7
Streptococcus_sanguinis_SK36_uid58381	96	4	48
Streptococcus_suis_05ZYH33_uid58663	97	-73	-3
Streptococcus_suis_98HAH33_uid58665	98	-67	-21
Streptococcus_suis_A7_uid162111	99	-37	165
Streptococcus_suis_BM407_uid59321	100	120	355
Streptococcus_suis_D12_uid162127	101	38	163
Streptococcus_suis_D9_uid162125	102	33	287
Streptococcus_suis_GZ1_uid161937	103	-34	6
Streptococcus_suis_JS14_uid162095	104	-19	151
Streptococcus_suis_P1_7_uid32235	105	72	216
Streptococcus_suis_S735_uid174333	106	-24	12
Streptococcus_suis_SC070731_uid193769	107	102	149
Streptococcus_suis_SC84_uid59323	108	83	238
Streptococcus_suis_SS12_uid162123	109	-64	30
Streptococcus_suis_ST1_uid167482	110	-9	213
Streptococcus_suis_ST3_uid66327	111	-8	144
Streptococcus_suis_T15_uid226112	112	3	102
Streptococcus_suis_TL13_uid203123	113	8	88
Streptococcus_suis_YB51_uid222230	114	-41	14
Streptococcus_thermophilus_CNRZ1066_uid58221	115	-15	-62
Streptococcus_thermophilus_JIM_8232_uid162157	116	-147	-77
Streptococcus_thermophilus_LMD_9_uid58327	117	245	666
Streptococcus_thermophilus_LMG_18311_uid58219	118	11	-44
Streptococcus_thermophilus_MN_ZLW_002_uid166827	119	38	234
Streptococcus_thermophilus_ND03_uid162015	120	22	220
Streptococcus_uberis_0140J_uid57959	121	45	147
	122	40,23140496	177,0082645

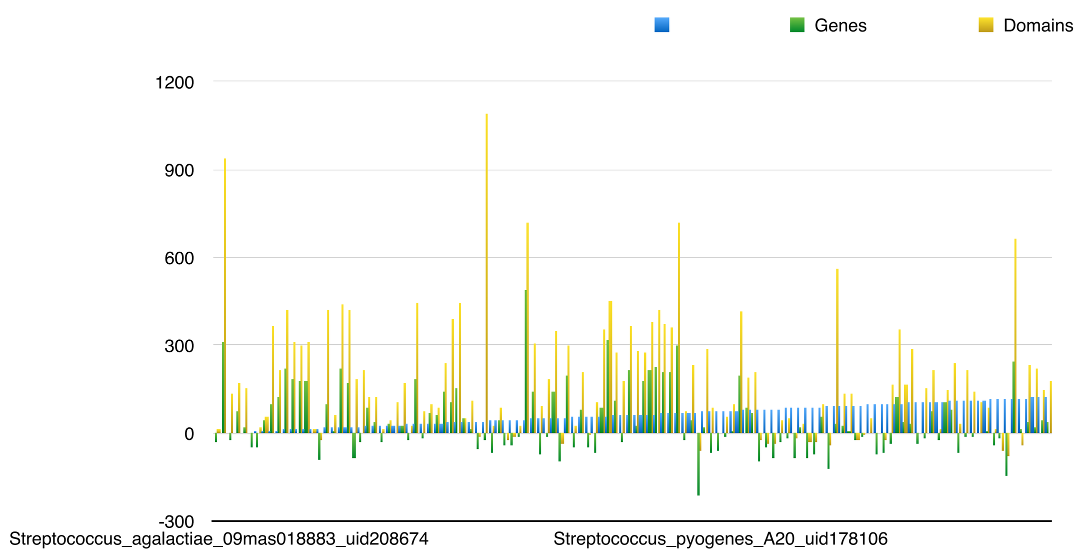

Table 1-1

StrainNumber	Genes	Domains
1	-32	12
2	309	940
3	-21	135
4	74	174
5	19	152
6	-50	-2
7	-47	20
8	46	56
9	96	368
10	120	216
11	220	421
12	185	314
13	177	299
14	178	312
15	3	16
16	-88	-24
17	101	420
18	8	64
19	222	438
20	171	418
21	-84	186
22	-31	215
23	89	125
24	36	122
25	-31	15
26	32	41
27	4	106

Table 1-1-1

StrainNumber	Genes	Domains
28	28	169
29	-21	28
30	186	447
31	-19	72
32	71	101
33	65	87
34	139	238
35	103	391
36	155	447
37	47	50
38	16	111
39	-51	-10
40	-23	1089
41	-63	23
42	42	89
43	-40	-24
44	-40	-8
45	-10	24
46	485	721
47	142	308

Table 1-1-1-2

StrainNumber	Genes	Domains
48	-69	90
49	-8	184
50	140	347
51	-99	-37
52	194	300
53	-47	25
54	81	210
55	-46	-5
56	-63	107
57	86	357
58	315	449
59	110	274
60	-27	177
61	213	369
62	28	281
63	178	278
64	215	379
65	225	423
66	210	372
67	211	363
68	298	717
69	-24	75
70	41	234
71	-212	-60
72	19	290
73	-67	84

Table 1-1-1-2-1

StrainNumber	Genes	Domains
74	-60	-1
75	-12	56
76	9	96
77	195	413
78	84	190
79	66	208
80	-97	-21
81	-47	-35
82	-85	-36
83	-32	45
84	-16	52
85	-83	-18
86	21	31
87	-81	-29
88	-70	-27
89	54	100
90	-121	-39
91	34	558
92	26	134

Table 1-1-1-2-1-1

StrainNumber	Genes	Domains
93	8	134
94	-23	-22
95	-8	-7
96	4	48
97	-73	-3
98	-67	-21
99	-37	165
100	120	355
101	38	163
102	33	287
103	-34	6
104	-19	151
105	72	216
106	-24	12
107	102	149
108	83	238
109	-64	30
110	-9	213
111	-8	144
112	3	102
113	8	88
114	-41	14
115	-15	-62
116	-147	-77
117	245	666
118	11	-44
119	38	234
120	22	220
121	45	147

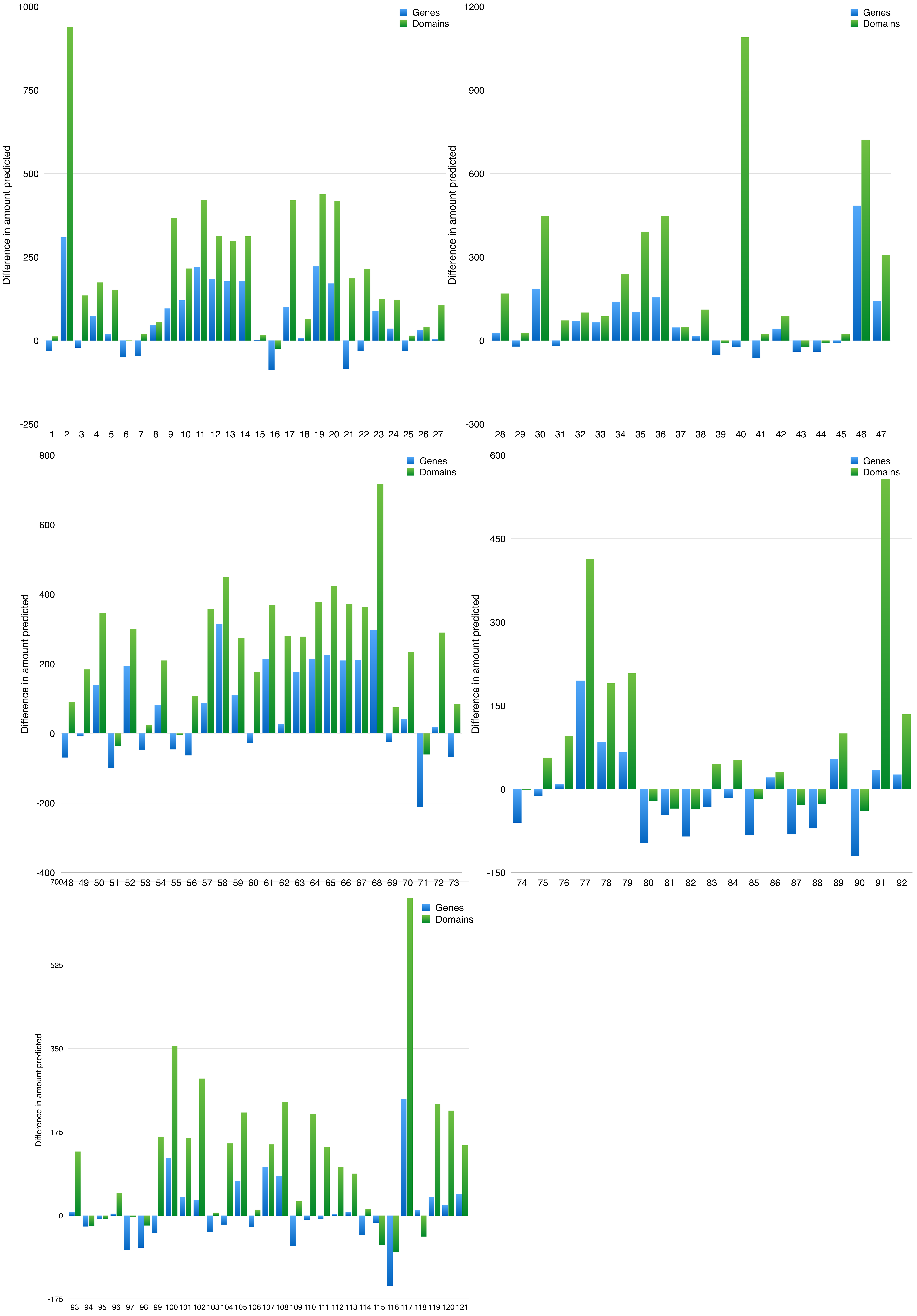

Genes
Domains
Difference in amount predicted
1000
750
500
250
0
-250
1 2 3 4 5 6 7 8 9 10 11 12 13 14 15 16 17 18 19 20 21 22 23 24 25 26 27
Genes
Domains
Difference in amount predicted
1200
900
600
300
0
-300
28 29 30 31 32 33 34 35 36 37 38 39 40 41 42 43 44 45 46 47
Genes
Domains
Difference in amount predicted
800
600
400
200
0
-200
-400
700
48 49 50 51 52 53 54 55 56 57 58 59 60 61 62 63 64 65 66 67 68 69 70 71 72 73
Genes
Domains
Difference in amount predicted
600
450
300
150
0
-150
74 75 76 77 78 79 80 81 82 83 84 85 86 87 88 89 90 91 92
Genes
Domains
Difference in amount predicted
525
350
175
0
-175
93 94 95 96 97 98 99 100 101 102 103 104 105 106 107 108 109 110 111 112 113 114 115 116 117 118 119 120 121

Strain	Number	Genes	Domains
670_6B	48	-69	90
70585	49	-8	184
A026	50	140	347
AP200	51	-99	-37
ATCC_700669	52	194	300
CGSP14	53	-47	25
D39	54	81	210
G54	55	-46	-5
gamPNI0373	56	-63	107
Hungary19A_6	57	86	357
INV104	58	315	449
INV200	59	110	274
JJA	60	-27	177
OXC141	61	213	369
P1031	62	28	281
R6	63	178	278
SPN034156	64	215	379
SPN034183	65	225	423
SPN994038	66	210	372
SPN994039	67	211	363
SPNA45	68	298	717
ST556	69	-24	75
Taiwan19F_14	70	41	234
TCH8431_19A	71	-212	-60
TIGR4	72	19	290

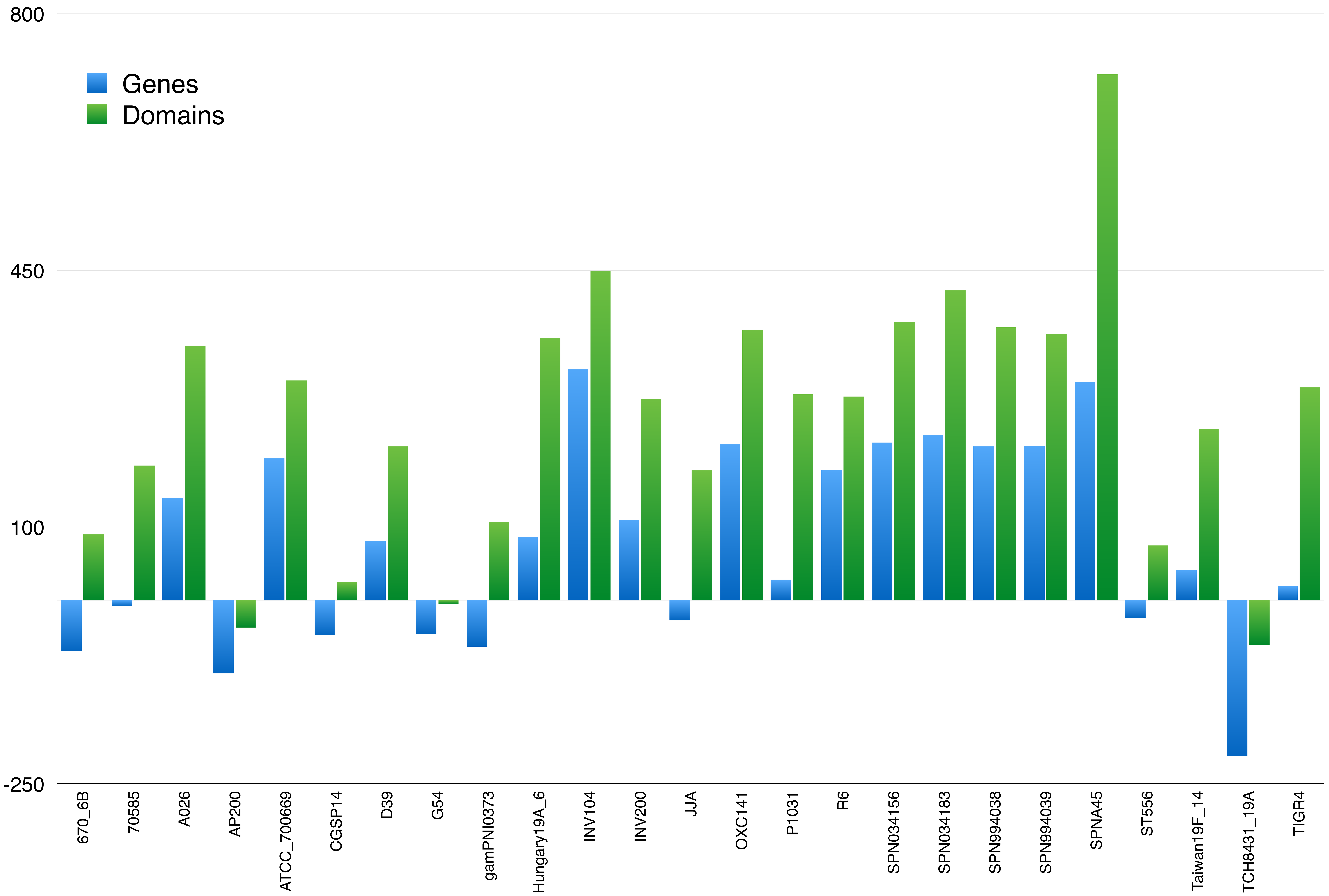
800
450
100
-250
Genes
Domains
670_6B
70585
A026
AP200
ATCC_700669
CGSP14
D39
G54
gamPNI0373
Hungary19A_6
INV104
INV200
JJA
OXC141
P1031
R6
SPN034156
SPN034183
SPN994038
SPN994039
SPNA45
ST556
Taiwan19F_14
TCH8431_19A
TIGR4

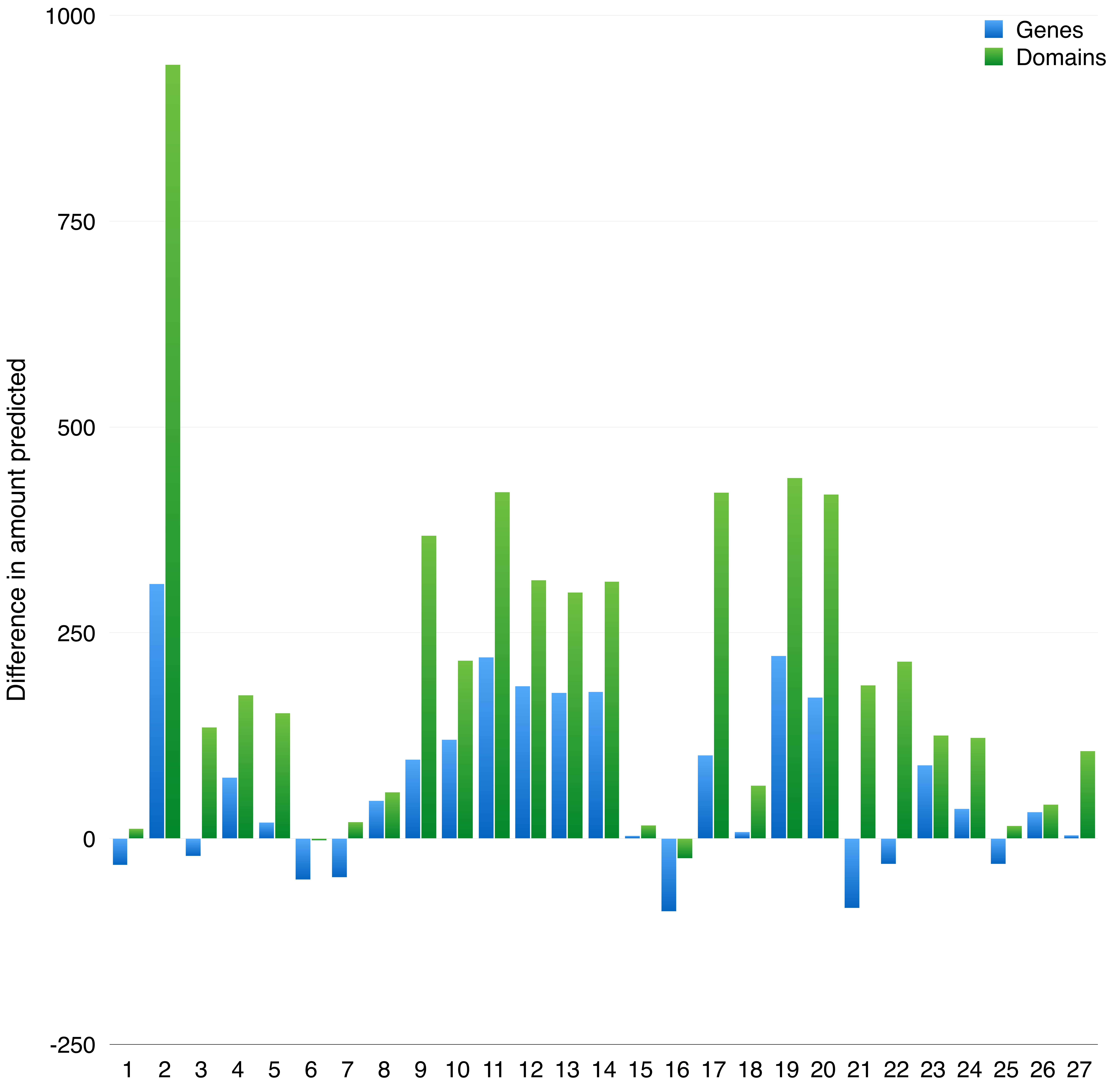
Genes
Domains
Difference in amount predicted
1000
750
500
250
0
-250
1
2
3
4
5
6
7
8
9
10
11
12
13
14
15
16
17
18
19
20
21
22
23
24
25
26
27

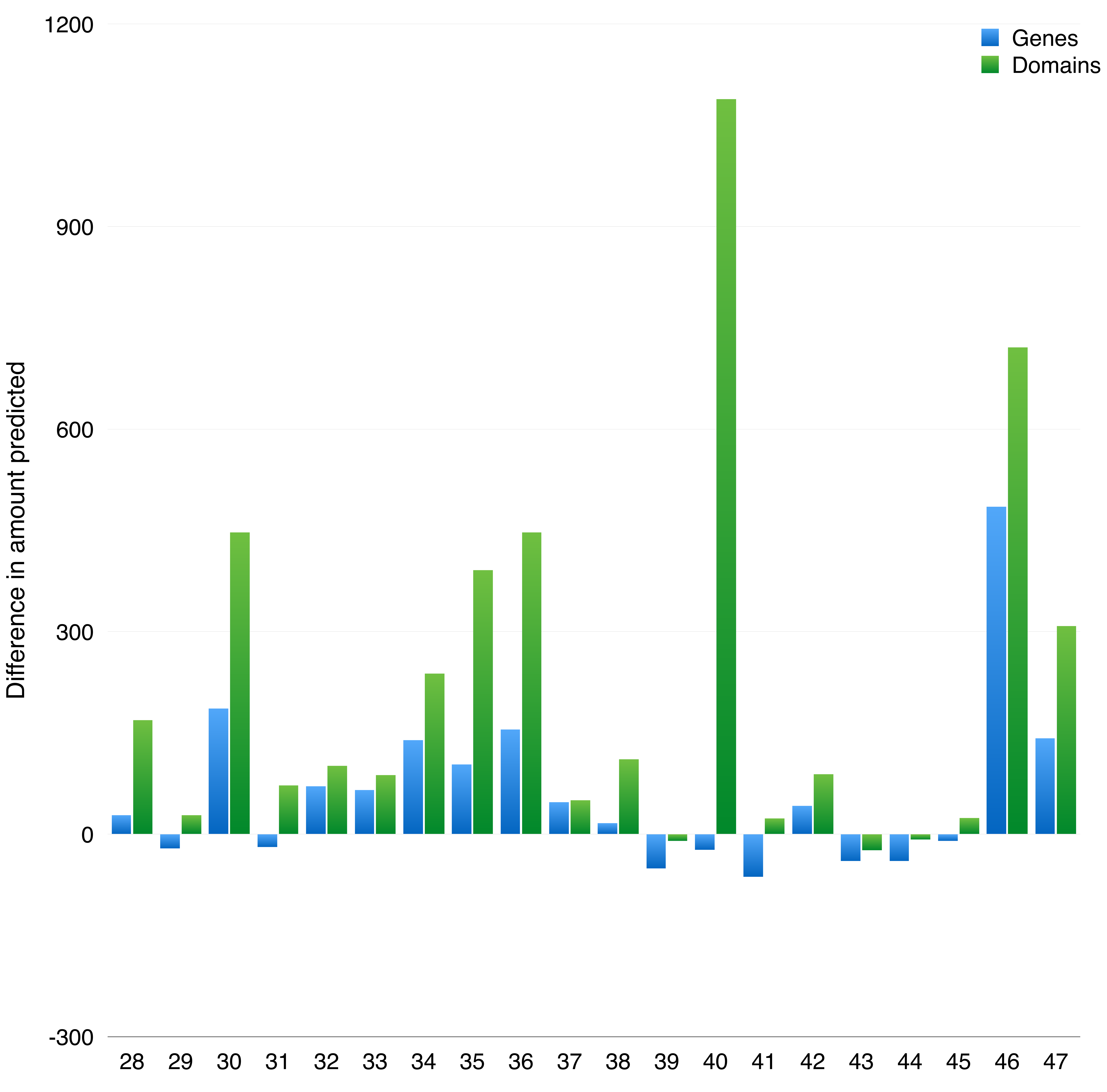

1200
900
600
300
0
-300
Difference in amount predicted
Genes
Domains
28
29
30
31
32
33
34
35
36
37
38
39
40
41
42
43
44
45
46
47

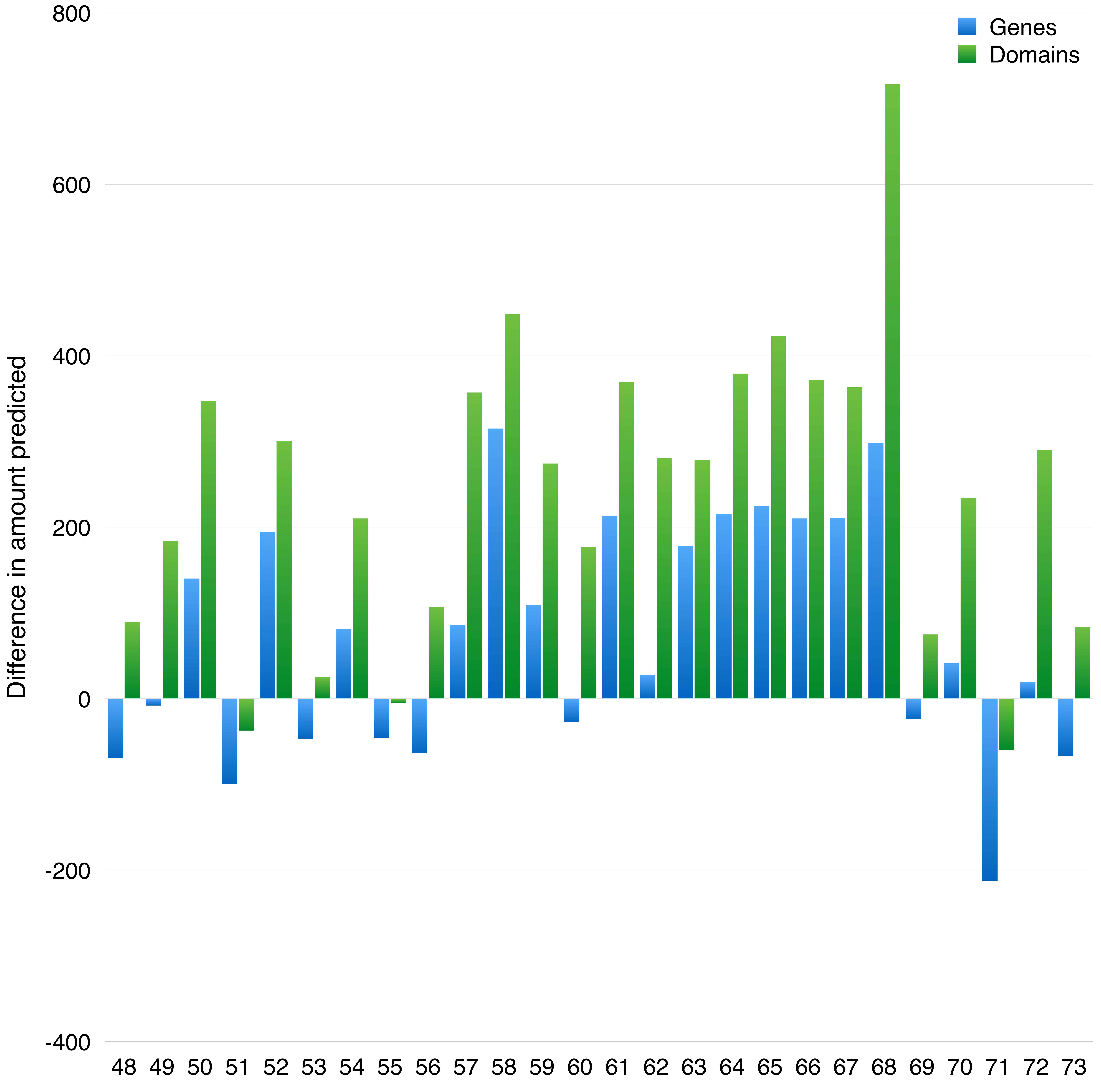

Genes
Domains
Difference in amount predicted
800
600
400
200
0
-200
-400
48 49 50 51 52 53 54 55 56 57 58 59 60 61 62 63 64 65 66 67 68 69 70 71 72 73

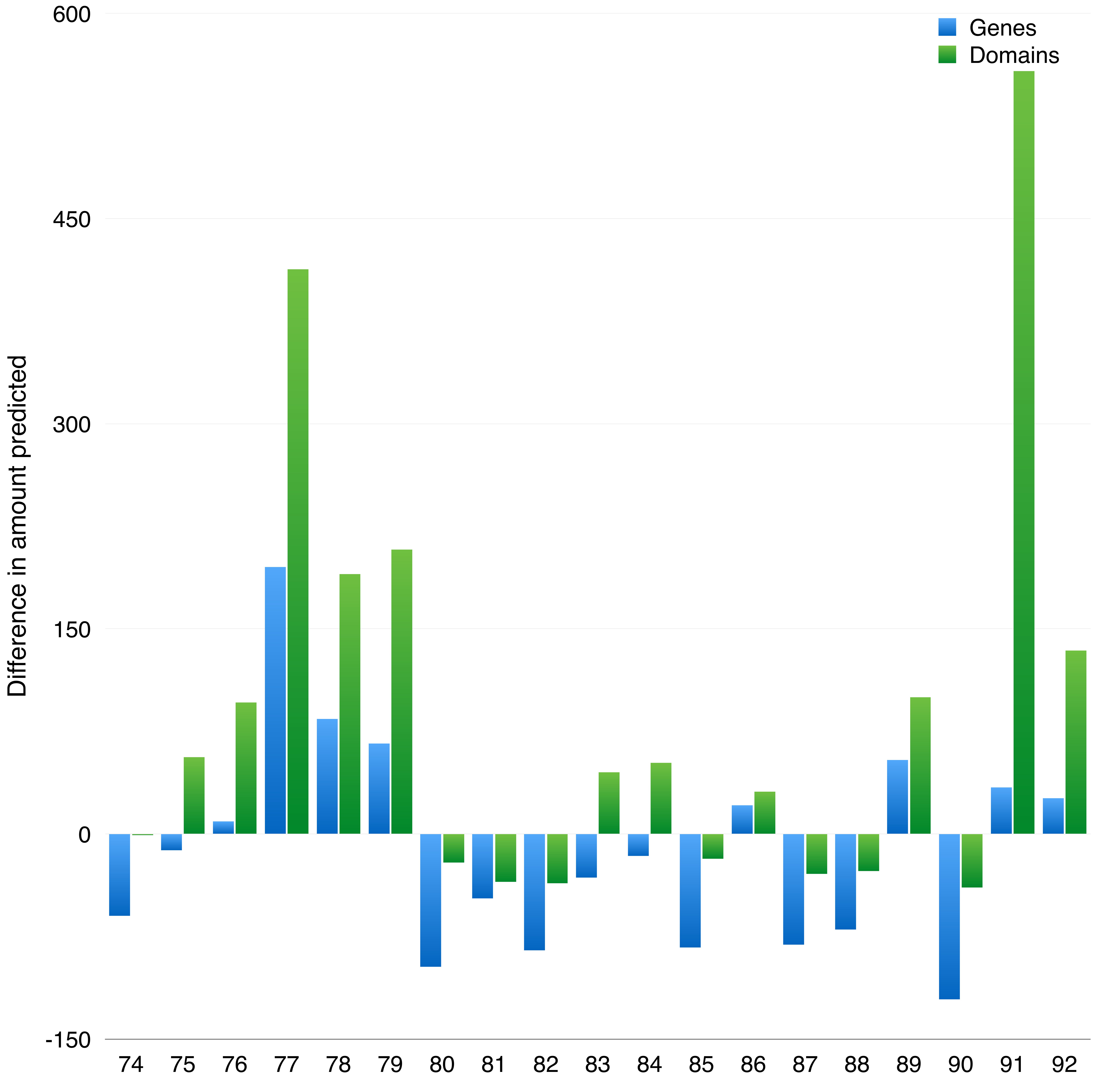

Genes
Domains
Difference in amount predicted
600
450
300
150
0
-150
74
75
76
77
78
79
80
81
82
83
84
85
86
87
88
89
90
91
92

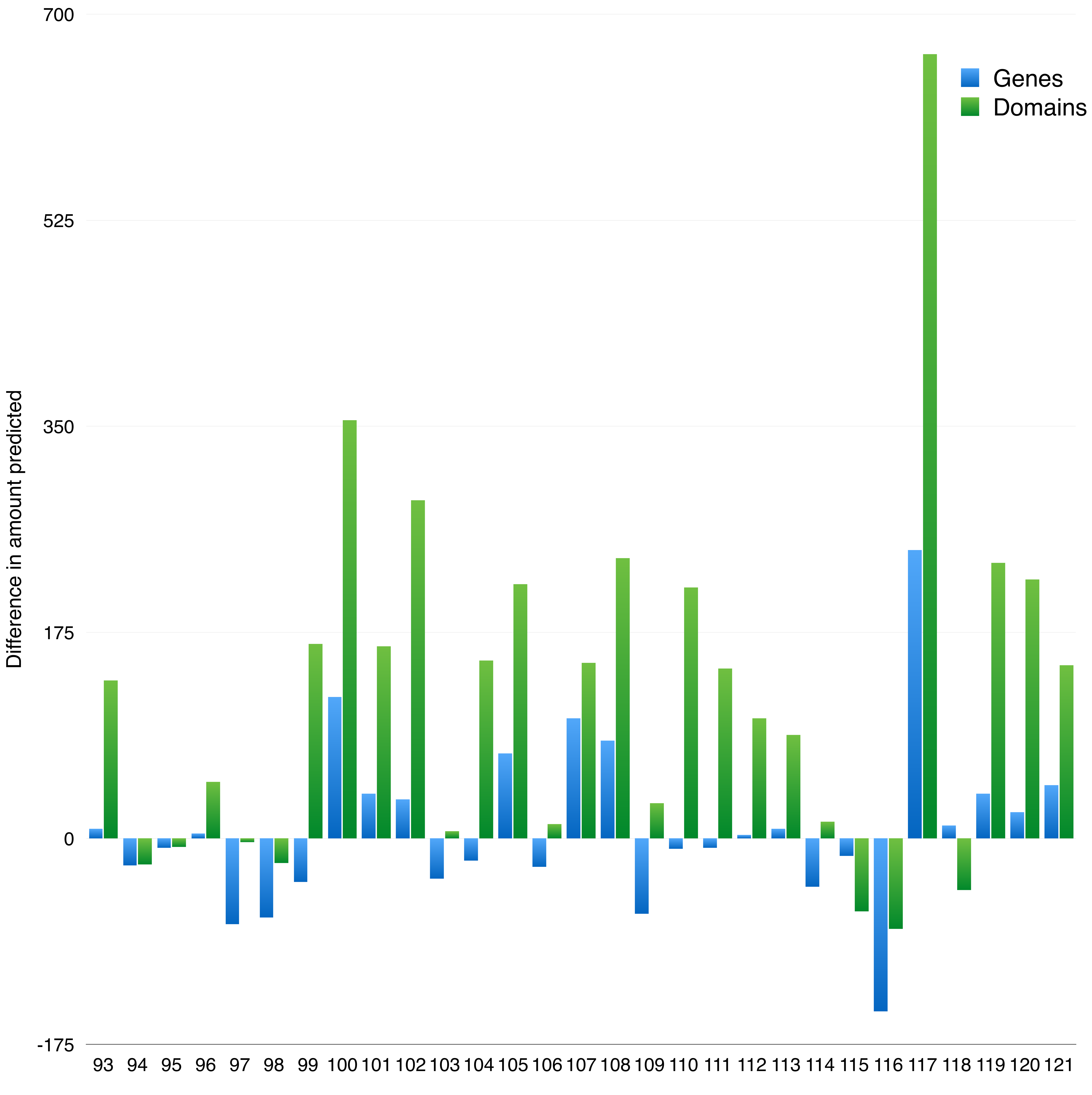
700
525
350
175
0
-175
Difference in amount predicted
Genes
Domains
93
94
95
96
97
98
99
100
101
102
103
104
105
106
107
108
109
110
111
112
113
114
115
116
117
118
119
120
121

Strain	Number	Genes	Domains
S.agalactiae 09mas018883 uid208674	1	-32	12
S.agalactiae 2 22 uid202215	2	309	940
S.agalactiae 2603V R uid57943	3	-21	135
S.agalactiae A909 uid57935	4	74	174
S.agalactiae GD201008 001 uid175780	5	19	152
S.agalactiae ILRI005 uid208676	6	-50	-2
S.agalactiae ILRI112 uid208675	7	-47	20
S.agalactiae NEM316 uid61585	8	46	56
S.agalactiae SA20 06 uid178722	9	96	368
S.anginosus C1051 uid218003	10	120	216
S.anginosus C238 uid218004	11	220	421
S.constellatus pharyngis C1050 uid218002	12	185	314
S.constellatus pharyngis C232 uid217998	13	177	299
S.constellatus pharyngis C818 uid218001	14	178	312
S.dysgalactiae equisimilis 167 uid222822	15	3	16
S.dysgalactiae equisimilis AC 2713 uid178644	16	-88	-24
S.dysgalactiae equisimilis ATCC 12394 uid161979	17	101	420
S.dysgalactiae equisimilis GGS 124 uid59103	18	8	64
S.dysgalactiae equisimilis RE378 uid176684	19	222	438
S.equi 4047 uid59259	20	171	418
S.equi zooepidemicus ATCC 35246 uid162155	21	-84	186
S.equi zooepidemicus MGCS10565 uid59263	22	-31	215
S.equi zooepidemicus uid59261	23	89	125
S.gallolyticus ATCC 43143 uid162103	24	36	122
S.gallolyticus ATCC BAA 2069 uid63617	25	-31	15
S.gallolyticus UCN34 uid46061	26	32	41
S.gordonii Challis substr CH1 uid57667	27	4	106
S.I G2 uid224251	28	28	169
S.I P16 uid224252	29	-21	28
S.infantarius CJ18 uid87033	30	186	447
S.iniae SF1 uid206041	31	-19	72
S.intermedius B196 uid218000	32	71	101
S.intermedius C270 uid217999	33	65	87
S.intermedius JTH08 uid168614	34	139	238
S.lutetiensis 033 uid213397	35	103	391
S.macedonicus ACA DC 198 uid81631	36	155	447
S.mitis B6 uid46097	37	47	50
S.mutans GS 5 uid169223	38	16	111
S.mutans LJ23 uid162197	39	-51	-10
S.mutans NN2025 uid46353	40	-23	1089
S.mutans UA159 uid57947	41	-63	23
S.oligofermentans AS 1 3089 uid201429	42	42	89
S.oralis Uo5 uid65449	43	-40	-24
S.parasanguinis ATCC 15912 uid49313	44	-40	-8
S.parasanguinis FW213 uid163997	45	-10	24
S.parauberis KCTC 11537 uid67355	46	485	721
S.pasteurianus ATCC 43144 uid68019	47	142	308
S.pneumoniae 670 6B uid52533	48	-69	90
S.pneumoniae 70585 uid59125	49	-8	184
S.pneumoniae A026 uid226114	50	140	347
S.pneumoniae AP200 uid52453	51	-99	-37
S.pneumoniae ATCC 700669 uid59287	52	194	300
S.pneumoniae CGSP14 uid59181	53	-47	25
S.pneumoniae D39 uid58581	54	81	210
S.pneumoniae G54 uid59167	55	-46	-5
S.pneumoniae gamPNI0373 uid175861	56	-63	107
S.pneumoniae Hungary19A 6 uid59117	57	86	357
S.pneumoniae INV104 uid162039	58	315	449
S.pneumoniae INV200 uid162035	59	110	274
S.pneumoniae JJA uid59121	60	-27	177
S.pneumoniae OXC141 uid162037	61	213	369
S.pneumoniae P1031 uid59123	62	28	281
S.pneumoniae R6 uid57859	63	178	278
S.pneumoniae SPN034156 uid197185	64	215	379
S.pneumoniae SPN034183 uid197186	65	225	423
S.pneumoniae SPN994038 uid197187	66	210	372

Strain	Number	Genes	Domains
S.pneumoniae SPN994039 uid197188	67	211	363
S.pneumoniae SPNA45 uid174986	68	298	717
S.pneumoniae ST556 uid162191	69	-24	75
S.pneumoniae Taiwan19F 14 uid59119	70	41	234
S.pneumoniae TCH8431 19A uid49735	71	-212	-60
S.pneumoniae TIGR4 uid57857	72	19	290
S.pseudopneumoniae IS7493 uid71153	73	-67	84
S.pyogenes A20 uid178106	74	-60	-1
S.pyogenes Alab49 uid162171	75	-12	56
S.pyogenes HSC5 uid212978	76	9	96
S.pyogenes M1 476 uid193766	77	195	413
S.pyogenes M1 GAS uid57845	78	84	190
S.pyogenes Manfredo uid57847	79	66	208
S.pyogenes MGAS10270 uid58571	80	-97	-21
S.pyogenes MGAS10394 uid58105	81	-47	-35
S.pyogenes MGAS10750 uid58575	82	-85	-36
S.pyogenes MGAS15252 uid158037	83	-32	45
S.pyogenes MGAS1882 uid158061	84	-16	52
S.pyogenes MGAS2096 uid58573	85	-83	-18
S.pyogenes MGAS315 uid57911	86	21	31
S.pyogenes MGAS5005 uid58337	87	-81	-29
S.pyogenes MGAS6180 uid58335	88	-70	-27
S.pyogenes MGAS8232 uid57871	89	54	100
S.pyogenes MGAS9429 uid58569	90	-121	-39
S.pyogenes NZ131 uid59035	91	34	558
S.pyogenes SSI 1 uid57895	92	26	134
S.salivarius 57 I uid162151	93	8	134
S.salivarius CCHSS3 uid70481	94	-23	-22
S.salivarius JIM8777 uid162145	95	-8	-7
S.sanguinis SK36 uid58381	96	4	48
S.suis 05ZYH33 uid58663	97	-73	-3
S.suis 98HAH33 uid58665	98	-67	-21
S.suis A7 uid162111	99	-37	165
S.suis BM407 uid59321	100	120	355
S.suis D12 uid162127	101	38	163
S.suis D9 uid162125	102	33	287
S.suis GZ1 uid161937	103	-34	6
S.suis JS14 uid162095	104	-19	151
S.suis P1 7 uid32235	105	72	216
S.suis S735 uid174333	106	-24	12
S.suis SC070731 uid193769	107	102	149
S.suis SC84 uid59323	108	83	238
S.suis SS12 uid162123	109	-64	30
S.suis ST1 uid167482	110	-9	213
S.suis ST3 uid66327	111	-8	144
S.suis T15 uid226112	112	3	102
S.suis TL13 uid203123	113	8	88
S.suis YB51 uid222230	114	-41	14
S.thermophilus CNRZ1066 uid58221	115	-15	-62
S.thermophilus JIM 8232 uid162157	116	-147	-77
S.thermophilus LMD 9 uid58327	117	245	666
S.thermophilus LMG 18311 uid58219	118	11	-44
S.thermophilus MN ZLW 002 uid166827	119	38	234
S.thermophilus ND03 uid162015	120	22	220
S.uberis 0140J uid57959	121	45	147
